# A Six Months Exercise Intervention Influences the Genome-wide DNA Methylation Pattern in Human Adipose Tissue

**DOI:** 10.1371/journal.pgen.1003572

**Published:** 2013-06-27

**Authors:** Tina Rönn, Petr Volkov, Cajsa Davegårdh, Tasnim Dayeh, Elin Hall, Anders H. Olsson, Emma Nilsson, Åsa Tornberg, Marloes Dekker Nitert, Karl-Fredrik Eriksson, Helena A. Jones, Leif Groop, Charlotte Ling

**Affiliations:** 1 Department of Clinical Sciences, Epigenetics and Diabetes, Lund University Diabetes Centre, CRC, Malmö, Sweden; 2 Department of Health Sciences, Division of Physiotherapy, Lund University, Lund, Sweden; 3 School of Medicine, Royal Brisbane Clinical School, The University of Queensland, Herston, Queensland, Australia; 4 Department of Clinical Sciences, Vascular Diseases, Lund University, Malmö, Sweden; 5 Department of Experimental Medical Science, Division of Diabetes, Metabolism and Endocrinology, Lund University, BMC C11, Lund, Sweden; 6 Department of Clinical Sciences, Diabetes and Endocrinology, Lund University Diabetes Centre, CRC, Malmö, Sweden; Albert Einstein College of Medicine, United States of America

## Abstract

Epigenetic mechanisms are implicated in gene regulation and the development of different diseases. The epigenome differs between cell types and has until now only been characterized for a few human tissues. Environmental factors potentially alter the epigenome. Here we describe the genome-wide pattern of DNA methylation in human adipose tissue from 23 healthy men, with a previous low level of physical activity, before and after a six months exercise intervention. We also investigate the differences in adipose tissue DNA methylation between 31 individuals with or without a family history of type 2 diabetes. DNA methylation was analyzed using Infinium HumanMethylation450 BeadChip, an array containing 485,577 probes covering 99% RefSeq genes. Global DNA methylation changed and 17,975 individual CpG sites in 7,663 unique genes showed altered levels of DNA methylation after the exercise intervention (*q*<0.05). Differential mRNA expression was present in 1/3 of gene regions with altered DNA methylation, including *RALBP1*, *HDAC4* and *NCOR2* (*q*<0.05). Using a luciferase assay, we could show that increased DNA methylation *in vitro* of the *RALBP1* promoter suppressed the transcriptional activity (*p* = 0.03). Moreover, 18 obesity and 21 type 2 diabetes candidate genes had CpG sites with differences in adipose tissue DNA methylation in response to exercise (*q*<0.05), including *TCF7L2* (6 CpG sites) and *KCNQ1* (10 CpG sites). A simultaneous change in mRNA expression was seen for 6 of those genes. To understand if genes that exhibit differential DNA methylation and mRNA expression in human adipose tissue *in vivo* affect adipocyte metabolism, we silenced *Hdac4* and *Ncor2* respectively in 3T3-L1 adipocytes, which resulted in increased lipogenesis both in the basal and insulin stimulated state. In conclusion, exercise induces genome-wide changes in DNA methylation in human adipose tissue, potentially affecting adipocyte metabolism.

## Author Summary

Given the important role of epigenetics in gene regulation and disease development, we here present the genome-wide DNA methylation pattern of 476,753 CpG sites in adipose tissue obtained from healthy men. Since environmental factors potentially change metabolism through epigenetic modifications, we examined if a six months exercise intervention alters the DNA methylation pattern as well as gene expression in human adipose tissue. Our results show that global DNA methylation changes and 17,975 individual CpG sites alter the levels of DNA methylation in response to exercise. We also found differential DNA methylation of 39 candidate genes for obesity and type 2 diabetes in human adipose tissue after exercise. Additionally, we provide functional proof that genes, which exhibit both differential DNA methylation and gene expression in human adipose tissue in response to exercise, influence adipocyte metabolism. Together, this study provides the first detailed map of the genome-wide DNA methylation pattern in human adipose tissue and links exercise to altered adipose tissue DNA methylation, potentially affecting adipocyte metabolism.

## Introduction

A sedentary lifestyle, a poor diet and new technologies that reduce physical activity cause health problems worldwide, as reduced energy expenditure together with increased energy intake lead to weight gain and increased cardiometabolic health risks [1]. Obesity is an important predictor for the development of both type 2 diabetes (T2D) and cardiovascular diseases, which suggests a central role for adipose tissue in the development of these conditions [2]. Adipose tissue is an endocrine organ affecting many metabolic pathways, contributing to total glucose homeostasis [2]. T2D is caused by a complex interplay of genetic and lifestyle factors [3], and a family history of T2D has been associated with reduced physical fitness and an increased risk of the disease [4]–[6]. Individuals with high risk of developing T2D strongly benefit from non-pharmacological interventions, involving diet and exercise [7], [8]. Exercise is important for physical health, including weight maintenance and its beneficial effects on triglycerides, cholesterol and blood pressure, suggestively by activating a complex program of transcriptional changes in target tissues.

Epigenetic mechanisms such as DNA methylation are considered to be important in phenotype transmission and the development of different diseases [9]. The epigenetic pattern is mainly established early in life and thereafter maintained in differentiated cells, but age-dependent alterations still have the potential to modulate gene expression and translate environmental factors into phenotypic traits [10]–[13]. In differentiated mammalian cells, DNA methylation usually occurs in the context of CG dinucleotides (CpGs) and is associated with gene repression [14]. Changes in epigenetic profiles are more common than genetic mutations and may occur in response to environmental, behavioural, psychological and pathological stimuli [15]. Furthermore, genetic variation not associated with a phenotype could nonetheless affect the extent of variability of that phenotype through epigenetic mechanisms, such as DNA methylation. It is not known whether epigenetic modifications contribute to the cause or transmission of T2D between generations. Recent studies in human skeletal muscle and pancreatic islets point towards the involvement of epigenetic modifications in the regulation of genes important for glucose metabolism and the pathogenesis of T2D [11], [12], [16]–[21]. However, there is limited information about the regulation of the epigenome in human adipose tissue [22].

The mechanisms behind the long-lasting effects of regular exercise are not fully understood, and most studies have focused on cellular and molecular changes in skeletal muscle. Recently, a global study of DNA methylation in human skeletal muscle showed changes in the epigenetic pattern in response to long-term exercise [23]. The aims of this study were to: 1) explore genome-wide levels of DNA methylation before and after a six months exercise intervention in adipose tissue from healthy, but previously sedentary men; 2) investigate the differences in adipose tissue DNA methylation between individuals with or without a family history of T2D; 3) relate changes in DNA methylation to adipose tissue mRNA expression and metabolic phenotypes *in vitro*.

## Results

### Baseline characteristics of individuals with (FH^+^) or without (FH^−^) a family history of type 2 diabetes

**Figure 1:**
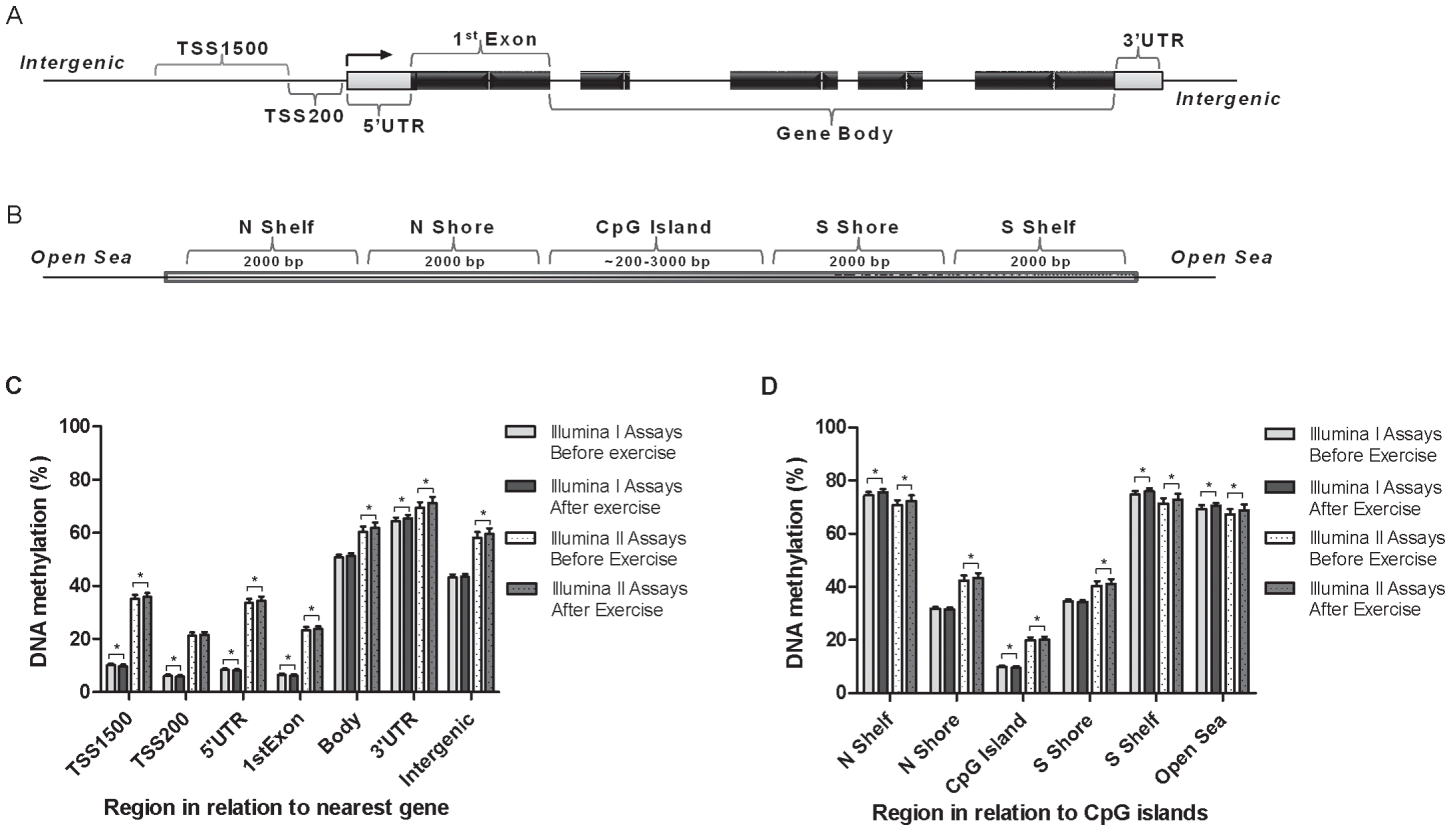
Location of analyzed CpG sites and global DNA methylation in human adipose tissue. All CpG sites analyzed on the Infinium HumanMethylation450 BeadChip are mapped to gene regions based on functional genome distribution (A) and to CpG island regions based on CpG content and neighbourhood context (B). In the lower panels, global DNA methylation in human adipose tissue is shown for each gene region (C) and for CpG island regions (D). Global DNA methylation is calculated as average DNA methylation based on all CpG sites in each region on the chip, and presented separately for Infinium I and Infinium II assays, respectively. Data is presented as mean ± SD. TSS, proximal promoter, defined as 200 bp (basepairs) or 1500 bp upstream of the transcription start site; UTR, untranslated region; CpG island, 200 bp (or more) stretch of DNA with a C+G content of 50% and an observed CpG/expected CpG in excess of 0.6; Shelf, regions flanking island shores, i.e., covering 2000–4000 bp distant from the CpG island; Shore: the flanking region of CpG islands, 0–2000 bp. *Significant difference between average DNA methylation before versus after exercise, *q*<0.05.

A total of 31 men, 15 FH^+^ and 16 FH^−^, had subcutaneous adipose tissue biopsies taken at baseline. The FH^+^ and FH^−^ individuals were group-wise matched for age, gender, BMI and VO_2max_ at inclusion, and there were no significant differences between FH^+^ and FH^−^ individuals, respectively (Table S1). DNA methylation in the adipose tissue was analyzed using the Infinium HumanMethylation450 BeadChip array. After quality control (QC), DNA methylation data was obtained for a total number of 476,753 sites. No individual CpG site showed a significant difference in DNA methylation between FH^+^ and FH^−^ men after false discovery rate (FDR) correction (*q*>0.05) [24]. Additionally, there were no global differences between the FH^+^ and FH^−^ individuals when calculating the average DNA methylation based on genomic regions (Figure 1a) or CpG content (Figure 1b; *q*>0.05).

### Clinical outcome and global changes in adipose tissue DNA methylation in response to exercise

Subcutaneous adipose tissue biopsies were taken from 23 men both before and after exercise, followed by successful DNA extraction and analysis of DNA methylation using the Infinium HumanMethylation450 BeadChip array. Since we found no significant differences in DNA methylation between FH^+^ and FH^−^ men at baseline, the two groups were combined when examining the impact of exercise on DNA methylation in adipose tissue. In Table 1 the clinical and metabolic outcomes of the exercise intervention are presented for these 23 men, showing a significant decrease in waist circumference, waist/hip ratio, diastolic blood pressure, and resting heart rate, whereas a significant increase was seen for VO_2max_ and HDL.

**Table 1:** Clinical characteristics of study participants (*n* = 23) with DNA methylation data both before (baseline) and after the exercise intervention.

Characteristics	Baseline	After exercise	*p*-value
Age (years)	37.3±4.4	-	
Weight (kg)	91.8±11.0	90.8±11.6	0.18
BMI (kg/m^2^)	28.2±2.9	27.9±3.1	0.18
Waist circumference (cm)	97.7±8.6	95.7±8.7	0.02
Waist/hip ratio	0.93±0.05	0.92±0.06	0.01
Fatmass (%)	22.8±6.0	23.1±6.6	0.59
Fasting glucose (mmol/L)	5.01±0.64	4.95±0.59	0.51
2 h OGTT glucose (mmol/L)	6.17±1.02	5.86±1.47	0.32
HbA1c (%)	4.31±0.31	4.31±0.34	1.00
Fasting insulin (µU/mL)	6.60±2.41	6.80±2.86	0.63
VO_2max_ (mL/kg/min)	33.1±4.6	36.2±6.2	0.003
Systolic BP (mmHg)	132.5±10.2	129.9±11.8	0.34
Diastolic BP (mmHg)	79.3±9.3	74.8±10.7	0.04
Pulse (beats/min)	73.9±10.6	67.3±11.2	0.03
Total cholesterol (mmol/L)	4.99±0.71	4.63±1.12	0.07
Triglycerides (mmol/L)	1.63±1.30	1.26±0.98	0.20
LDL (mmol/L)	3.36±0.63	3.24±0.63	0.41
HDL (mmol/L)	1.04±0.21	1.11±0.21	0.02
LDL/HDL	3.31±0.89	3.02±0.92	0.053

Data are expressed as mean ± SD, based on paired t-tests and two-tailed *p*-values. BP, blood pressure; LDL, low density lipoprotein; HDL, high density lipoprotein.

To evaluate the global human methylome in adipose tissue, we first calculated the average level of DNA methylation in groups based on either the functional genome distribution (Figure 1a), or the CpG content and neighbourhood context (Figure 1b). We also present the average level of DNA methylation separately for the Infinium I (*n* = 126,804) and Infinium II (*n* = 326,640) assays due to different β-value distributions for these assays [25]. When evaluating Infinium I assays in relation to nearest gene, the global level of DNA methylation after exercise increased in the 3′ untranslated region (UTR; *q*<0.05), whereas a decrease was seen in the region 1500–200 bp upstream of transcription start (TSS1500), TSS200, 5′UTR and within the first exon (1st Exon; *q*<0.05). The global DNA methylation level of Infinium II assays increased significantly (*q*<0.05) after exercise within all regions except TSS200 (Figure 1c and Table S2). In general, the average level of DNA methylation was low in the region from TSS1500 to the 1st Exon (5–36%), whereas the gene body, the 3′UTR and intergenic region displayed average DNA methylation levels ranging from 43–72% (Figure 1c and Table S2). When evaluating global DNA methylation based on CpG content and distance to CpG islands, average DNA methylation for Infinium I assays decreased significantly after exercise in CpG islands, whereas an increase was seen in northern and southern shelves (regions 2000–4000 bp distant from CpG islands) as well as in the open sea (regions further away from a CpG island) (*q*<0.05; Figure 1d and Table S2). For Infinium II assays, average DNA methylation was significantly increased in all regions after the exercise intervention (*q*<0.05; Figure 1d and Table S2). The global level of DNA methylation was low within CpG islands (9–21%), intermediate within the shores (2000 bp regions flanking the CpG islands; 31–44%), whereas the shelves and the open sea showed the highest level of DNA methylation (67–76%; Figure 1d and Table S2). Although technical variation between probe types has been reported for the Infinium HumanMethylation450 BeadChip array, seen as a divergence between the β-values distribution retrieved from the Infinium I and II assays [25], the global differences in DNA methylation we observe between probe types are more likely a result of skewed GC content due to the design criteria of the two different assays. Infinium I assays have significantly more CpGs within the probe body than the Infinium II assays, and 57% are annotated to CpG islands, whereas most Infinium II assays have less than three underlying CpGs in the probe and only 21% are designated as CpG islands [26].

### DNA methylation of individual CpG sites in human adipose tissue is influenced by exercise

**Figure 2:**
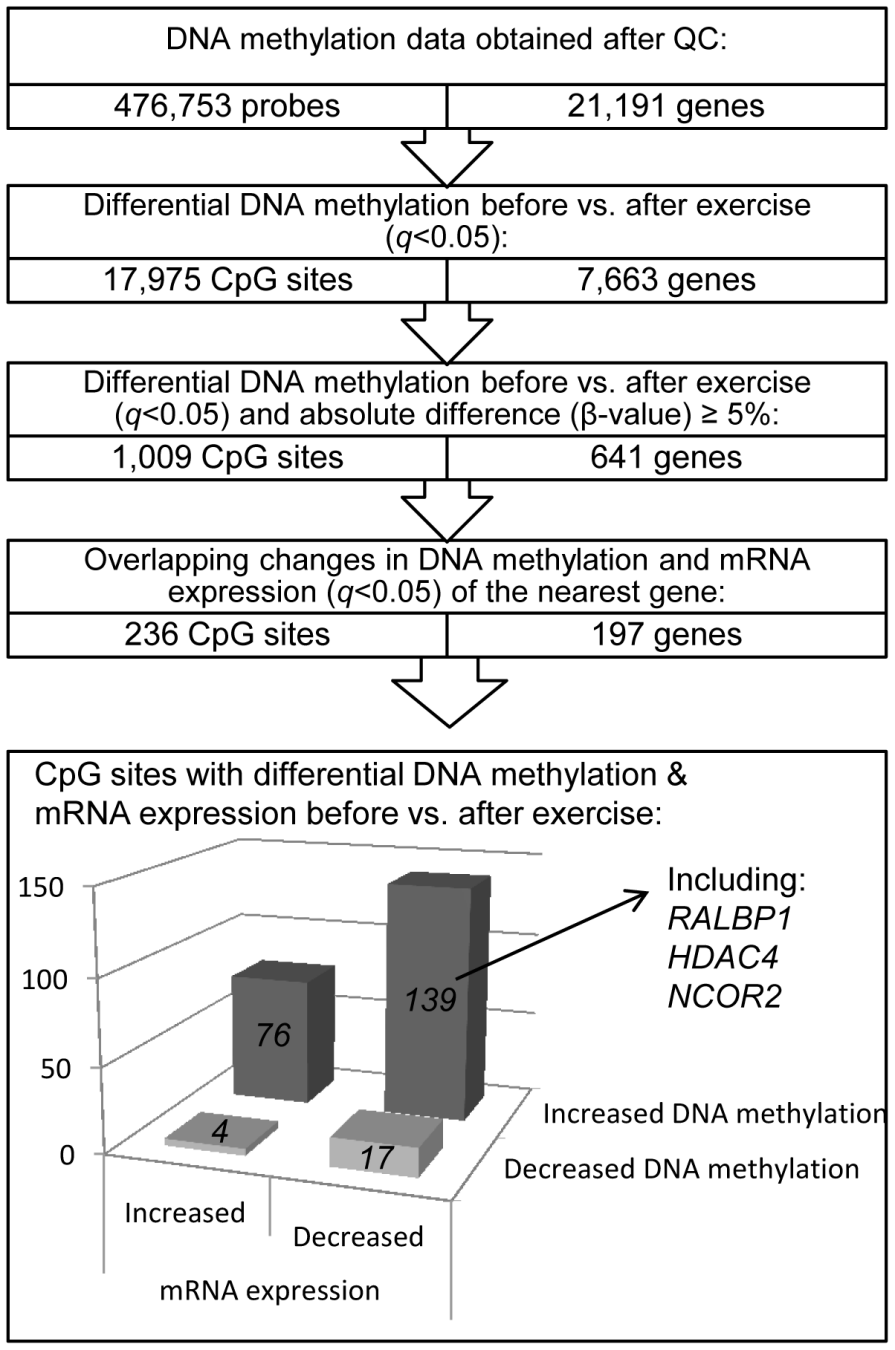
Analysis flowchart.

**Figure 3:**
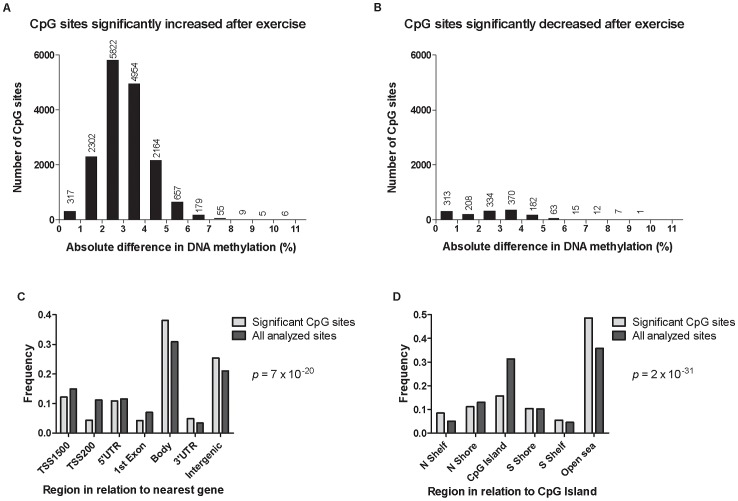
DNA methylation of individual CpG sites. The absolute change in DNA methylation of individual CpG sites with a significant difference after exercise compared with baseline (*q*<0.05) ranges from 0.2–10.9% (A and B). A) Number of sites with increased methylation in adipose tissue in response to exercise (*n* = 16,470). B) Number of sites with decreased DNA methylation in adipose tissue in response to exercise (*n* = 1,505). Panels C and D show the distribution of CpG sites with a significant change (*q*<0.05) and an absolute difference ≥5% in DNA methylation in adipose tissue before versus after exercise, in comparison to all analyzed sites on the Infinium HumanMethylation450 BeadChip. C) Distribution of significant CpG sites vs. all analyzed sites in relation to nearest gene regions. D) Distribution of significant CpG sites vs. all analyzed sites in relation to CpG island regions. *The overall distribution of significant CpG sites compared with all analyzed sites on the Infinium HumanMethylation450 BeadChip was analyzed using a chi^2^ test.

We next investigated if there was a difference in DNA methylation in any of the 476,753 analyzed individual CpG sites in adipose tissue in response to exercise. A flowchart of the analysis process is found in Figure 2. SNPs within the probe were not a criterion for exclusion in this analysis, as the participants are their own controls, thereby excluding genetic variation within the tested pairs. Applying FDR correction (*q*<0.05) resulted in 17,975 CpG sites, corresponding to 7,663 unique genes, that exhibit differential DNA methylation in adipose tissue after exercise. Among these 17,975 individual sites, 16,470 increased and 1,505 decreased the level of DNA methylation in response to exercise, with absolute changes in DNA methylation ranging from 0.2–10.9% (Figure 3a–b). Aiming for biological relevance, we further filtered our results requiring the average change in DNA methylation (β-value) for each CpG site to be ≥5% before vs. after exercise. Adding the criteria with a ≥5% change in DNA methylation resulted in 1,009 significant individual CpG sites: 911 with increased and 98 with decreased levels of DNA methylation in response to the six months exercise intervention. Of those, 723 sites are annotated to one or more genes, and correspond to 641 unique gene IDs. A comparison of our 1,009 significant CpG sites with Infinium probes reported to cross-react to alternative genomic locations [27] showed only one probe with 50 bases and 14 probes with 49 bases matching to an alternative genomic location. Data of the most significant CpG sites (*q*<0.005) and the sites that exhibit the greatest change in adipose tissue DNA methylation (difference in DNA methylation >8%) in response to exercise are presented in Table 2–3 and included *ITPR2* and *TSTD1* for increased, and *LTBP4* for decreased DNA methylation. We found 7 CpG sites in this list to be targeted by Infinium probes reported to cross-react to alternative genomic locations (47 or 48 bases) [27]. Additionally, to investigate the possibility that the changes we see in response to exercise is rather an effect of epigenetic drift over time, we compared our 1,009 differentially methylated CpG sites (*q*<0.05, difference in β-value>5%) with three studies reporting aging-differentially methylated regions (a-DMRs) in a total of 597 unique positions [28]–[30]. Secondly we tested for association between age and the level of DNA methylation in the 31 individuals included at baseline in this study, representing a more valid age range (30–45 years) and tissue for the current hypothesis. We found no overlap between previously published a-DMRs or the age-associated CpG sites within our study (18 CpG sites; *p*<1×10^−5^), and the CpG sites differentially methylated after the exercise intervention.

**Table 2:** Changes in adipose tissue DNA methylation in response to a 6 months exercise intervention. Most significant CpG sites (*q*<0.005) with a difference in DNA methylation ≥5%.

	*Location in relation to*				*DNA Methylation (%)*					
Probe ID	Chr	Nearest Gene	Gene region	CpG Island	Before exercise	After exercise	Difference	*p*-value	*q*-value *(<0.005)*	Cross-reactive probes
cg04090794	1	*HSP90B3P*	TSS1500	Open sea	31.4±5.1	36.6±4.6	5.2	2.38×10^−7^	0.004	
cg05091570	1	*NAV1*	Body	CpG Island	30.9±4.1	37.0±3.5	6.1	4.77×10^−7^	0.004	
cg01828733	1	*NAV1*	TSS200;Body	CpG Island	40.6±4.1	46.2±4.3	5.5	1.19×10^−6^	0.004	
cg24553673	1	*NR5A2*	Body	S Shore	33.1±4.7	39.9±3.8	6.8	2.38×10^−7^	0.004	
cg27183818	1		Intergenic	Open sea	66.7±4.7	60.7±4.3	−6.0	7.15×10^−7^	0.004	
cg26091021	2		Intergenic	N Shelf	38.9±3.6	45.4±3.3	6.5	2.38×10^−7^	0.004	
cg26297203	2		Intergenic	N Shelf	52.5±3.2	57.6±3.0	5.0	1.19×10^−6^	0.004	
cg14091208	3	*CCDC48*	Body	CpG Island	41.4±4.6	47.3±4.8	5.9	1.19×10^−6^	0.004	
cg09217023	3		Intergenic	Open sea	57.2±4.0	62.6±3.2	5.5	2.38×10^−7^	0.004	
cg09380805	3		Intergenic	N Shelf	29.0±4.0	35.7±3.8	6.6	7.15×10^−7^	0.004	
cg17103081	4	*GPR125*	Body	N Shelf	63.4±5.2	68.4±4.9	5.1	1.19×10^−6^	0.004	
cg15133208	4	*SNCA*	5′UTR	N Shore	36.5±4.8	42.4±4.8	6.0	1.67×10^−6^	0.004	
cg14348967	4		Intergenic	Open sea	31.9±5.2	37.5±4.4	5.6	1.67×10^−6^	0.004	
cg21817858	5		Intergenic	CpG Island	46.2±5.2	51.8±4.4	5.6	2.38×10^−7^	0.004	
cg20934416	5		Intergenic	Open sea	76.4±4.9	81.7±3.4	5.3	2.38×10^−6^	0.005	
cg14246190	6	*EHMT2*	Body	N Shelf	65.1±4.3	70.4±3.4	5.3	2.38×10^−6^	0.005	
cg20284982	6	*IER3*	TSS1500	S Shore	45.3±5.5	51.2±3.6	5.9	2.38×10^−6^	0.005	48
cg12586150	6	*SERPINB1*	Body	N Shore	51.9±5.2	58.4±4.7	6.5	2.38×10^−6^	0.005	
cg09871057	7	*STX1A*	Body	CpG Island	52.3±3.5	57.4±3.2	5.1	1.19×10^−6^	0.004	
cg18550262	7		Intergenic	Open sea	39.5±3.4	45.0±3.2	5.5	2.38×10^−7^	0.004	
cg00555695	8	*PVT1*	Body	Open sea	40.3±3.9	45.8±3.4	5.5	2.38×10^−7^	0.004	48
cg13832372	9	*LHX6*	Body	S Shore	25.8±4.5	31.1±5.4	5.4	2.38×10^−6^	0.005	
cg02725718	10	*ENKUR*	Body	Open sea	65.6±3.8	70.8±3.1	5.2	2.38×10^−6^	0.005	47
cg12127706	11	*CTTN*	Body	Open sea	54.3±3.9	59.5±3.7	5.2	1.19×10^−6^	0.004	
cg02093168	11	*HCCA2*	Body	Open sea	61.2±5.8	67.5±4.2	6.4	1.19×10^−6^	0.004	47
cg22041190	11	*PKNOX2*	5′UTR	S Shore	36.0±4.5	41.0±4.1	5.0	1.67×10^−6^	0.004	
cg12439006	11		Intergenic	Open sea	64.5±4.2	69.7±3.2	5.2	2.38×10^−7^	0.004	
cg19896824	11		Intergenic	Open sea	53.8±5.4	60.6±4.1	6.9	2.38×10^−7^	0.004	
cg21999471	11		Intergenic	Open sea	41.1±5.3	46.7±3.6	5.6	2.38×10^−6^	0.005	
cg26828839	12	*ANO2*	Body	Open sea	32.5±5.3	39.7±5.5	7.1	1.19×10^−6^	0.004	
cg13203394	12	*ITPR2*	Body	Open sea	56.8±4.4	63.3±3.2	6.5	4.77×10^−7^	0.004	
cg26119796	13	*RB1*	Body	S Shore	57.0±4.8	62.4±4.5	5.4	1.67×10^−6^	0.004	47
cg00808648	14	*PACS2*	TSS1500	N Shore	44.0±4.1	49.3±4.1	5.3	4.77×10^−7^	0.004	
cg22396498	15	*CRTC3*	Body	Open sea	59.5±4.5	64.6±5.1	5.1	1.19×10^−6^	0.004	
cg07299078	16	*KIFC3*	Body;5′UTR	Open sea	49.6±4.3	55.9±4.9	6.4	2.38×10^−7^	0.004	48
cg05797594	16	*MIR1910;C16orf74*	TSS1500;5′UTR	Open sea	51.5±5.1	57.2±2.9	5.6	2.38×10^−6^	0.005	47
cg05516390	16	*ZFHX3*	5′UTR	N Shelf	41.8±4.4	49.8±4.4	8.0	1.19×10^−6^	0.004	
cg06078469	17	*MSI2*	Body	S Shore	43.5±3.6	48.8±4.2	5.4	4.77×10^−7^	0.004	
cg22386583	17	*RPTOR*	Body	Open sea	51.2±3.8	57.0±3.5	5.8	4.77×10^−7^	0.004	
cg11225357	17		Intergenic	Open sea	45.1±4.1	50.6±3.9	5.5	1.19×10^−6^	0.004	
cg20811236	18		Intergenic	N Shore	60.9±5.3	68.2±4.9	7.3	4.77×10^−7^	0.004	
cg21685776	18		Intergenic	S Shore	51.4±4.4	56.6±4.8	5.2	7.15×10^−7^	0.004	
cg21520111	19	*TRPM4*	Body	CpG Island	53.4±3.4	59.0±4.0	5.5	4.77×10^−7^	0.004	
cg21427956	20	*C20orf160*	3′UTR	S Shore	37.5±3.8	43.1±4.3	5.6	1.19×10^−6^	0.004	
cg08587504	20	*LOC647979*	TSS1500	S Shore	62.7±3.4	68.0±3.0	5.3	2.38×10^−6^	0.005	
cg10854441	22	*MLC1*	TSS1500	N Shelf	51.3±4.9	57.1±4.3	5.9	1.67×10^−6^	0.004	
cg04065832	X	*CDX4*	1stExon	CpG Island	50.8±4.4	56.8±4.6	6.0	2.38×10^−6^	0.005	
cg19926635	X	*KCND1*	3′UTR	S Shelf	49.8±4.4	55.2±3.9	5.4	1.19×10^−6^	0.004	

Data are presented as mean ± SD, based on paired non-parametric test and two-tailed *p*-values. Cross-reactive probes: Maximum number of bases (≥47) matched to cross-reactive target as reported by Chen et al. [27].

**Table 3:** Changes in adipose tissue DNA methylation in response to a 6 months exercise intervention. Significant CpG sites (*q*<0.05) with the biggest change in DNA methylation (>8%).

	*Location in relation to*				*DNA Methylation (%)*					
Probe ID	Chr	Nearest Gene	Gene	CpG Island	Before exercise	After exercise	Difference (>8%)	*p*-value	*q*-value	Cross-reactive probes
cg06550177	7		Intergenic	S Shore	29.6±7.2	40.6±7.8	10.9	1.67×10^−5^	0.008	
cg13906823	1	*TSTD1*	TSS200	CpG Island	39.2±12.5	50.1±15.6	10.9	4.03×10^−5^	0.011	
cg23397147	17		Intergenic	Open sea	48.1±11.0	58.9±7.5	10.8	4.75×10^−4^	0.028	
cg24161057	1	*TSTD1*	TSS200	CpG Island	35.9±13.5	46.6±14.6	10.7	2.10×10^−5^	0.009	
cg26155520	1		Intergenic	Open sea	55.6±7.1	66.0±6.6	10.4	7.87×10^−6^	0.007	
cg05874882	4		Intergenic	N Shore	34.0±9.1	44.2±6.7	10.1	6.03×10^−5^	0.013	
cg00257920	1		Intergenic	S Shelf	47.5±9.7	57.5±7.7	10.0	1.53×10^−4^	0.018	
cg03878654	16	*ZFHX3*	5′UTR	N Shore	56.6±6.7	65.9±6.9	9.3	1.81×10^−4^	0.019	
cg08360726	19	*PLD3*	5′UTR	CpG Island	29.7±8.0	38.9±11.8	9.2	1.28×10^−3^	0.043	
cg26682335	17	*ABR*	Body	Open sea	60.6±9.4	69.7±7.0	9.1	2.53×10^−4^	0.022	
cg01425666	7		Intergenic	CpG Island	33.3±6.8	42.3±5.7	9.0	2.62×10^−5^	0.010	
cg01750221	12		Intergenic	Open sea	52.3±7.5	61.1±6.4	8.8	8.49×10^−4^	0.036	
cg05455393	X	*FHL1*	TSS1500	N Shore	52.5±8.4	61.1±7.2	8.6	1.28×10^−4^	0.017	
cg22828884	3	*FOXP1*	Body	Open sea	62.6±4.4	71.2±4.3	8.6	1.67×10^−5^	0.008	
cg11837417	19	*CLDND2*	TSS1500	S Shore	65.3±6.4	73.9±5.2	8.6	4.08×10^−4^	0.027	
cg10323490	2	*THNSL2*	TSS1500	N Shore	64.1±8.1	72.6±6.2	8.5	9.76×10^−4^	0.038	
cg03934443	10		Intergenic	Open sea	67.4±11.8	75.8±5.6	8.4	9.76×10^−4^	0.038	
cg01775802	14	*RGS6*	Body	Open sea	63.2±10.1	71.4±10.9	8.2	9.76×10^−4^	0.038	
cg24606240	1	*NUCKS1*	TSS1500	S Shore	55.4±7.9	63.6±5.9	8.2	7.38×10^−4^	0.034	
cg23499846	17	*KIAA0664*	5′UTR	S Shore	54.0±5.9	62.0±4.3	8.0	1.03×10^−5^	0.007	
cg21821308	2	*ASAP2*	Body	CpG Island	42.0±8.5	33.8±5.9	−8.1	3.49×10^−4^	0.025	
cg19219423	10	*PRKG1*	Body	Open sea	55.4±7.7	47.1±6.8	−8.3	1.81×10^−4^	0.019	
cg03862437	3	*TMEM44*	Body	N Shore	46.3±7.0	38.0±5.2	−8.3	5.96×10^−6^	0.006	
cg08368520	7	*FOXK1*	Body	Open sea	52.9±7.8	44.5±8.0	−8.4	9.76×10^−4^	0.038	
cg01275887	7	*FOXK1*	Body	Open sea	66.3±8.5	57.7±6.6	−8.5	7.38×10^−4^	0.034	
cg06443678	17		Intergenic	Open sea	51.7±8.2	43.0±6.7	−8.7	2.98×10^−4^	0.024	
cg02514003	2		Intergenic	Open sea	70.6±6.5	61.7±8.6	−8.9	2.53×10^−4^	0.022	
cg26504110	19	*LTBP4*	Body	CpG Island	36.9±8.7	27.4±5.1	−9.5	2.98×10^−4^	0.024	

Data are presented as mean ± SD, based on paired non-parametric test and two-tailed *p*-values. Cross-reactive probes: Maximum number of bases (≥47) matched to cross-reactive target as reported by Chen et al. [27].

The genomic distribution of individual CpG sites with a significant change in DNA methylation ≥5% with exercise is shown in Figure 3c–d, in comparison to all probes located on the Infinium HumanMethylation450 BeadChip and passing QC. The distribution is based on location in relation to the functional genome distribution (Figure 3c) or CpG content and distance to CpG islands (Figure 3d). We found that the CpG sites with altered level of DNA methylation in response to exercise were enriched within the gene body and in intergenic regions, while the proximal promoter, in particular TSS200 and the 1st exon, had a low proportion of differentially methylated CpG sites (*p* = 7×10^−20^; Figure 3c). In relation to CpG content and distance to CpG islands, the region with the highest proportion of significant CpG sites compared to the distribution on the array was in the open sea, *i.e.*, regions more distant from a CpG island than 4000 bp. In contrast, the number of significant CpG sites found within the CpG islands was only half of what would be expected (*p* = 2×10^−31^; Figure 3d).

### Exercise induces overlapping changes in DNA methylation and mRNA expression

An increased level of DNA methylation has previously been associated with transcription repression [14]. We therefore related changes in adipose tissue DNA methylation of individual CpG-sites (*q*<0.05 and difference in mean β-values ≥5%) with changes in mRNA expression of the same gene (*q*<0.05) in response to exercise (Figure 2). We identified 236 CpG sites in 197 individual gene regions that exhibit differential DNA methylation together with a significant change in adipose tissue mRNA expression of the corresponding gene after exercise. Of these, 143 CpG sites (61%) connected to 115 genes showed an inverse relation to mRNA expression. After exercise, 139 CpG sites showed an increase in DNA methylation and a corresponding decrease in mRNA expression, including a gene for one of the GABA receptors (*GABBR1*), several genes encoding histone modifying enzymes (*EHMT1*, *EHMT2* and *HDAC4*) and a transcriptional co-repressor (*NCOR2*). Only four CpG sites were found to decrease in the level of DNA methylation with a concomitant increase in mRNA expression. Table S3 shows all significant results of DNA methylation sites with an inverse relation to mRNA expression in human adipose tissue before vs. after exercise.

### DNA methylation *in vitro* decreases reporter gene expression

**Figure 4:**
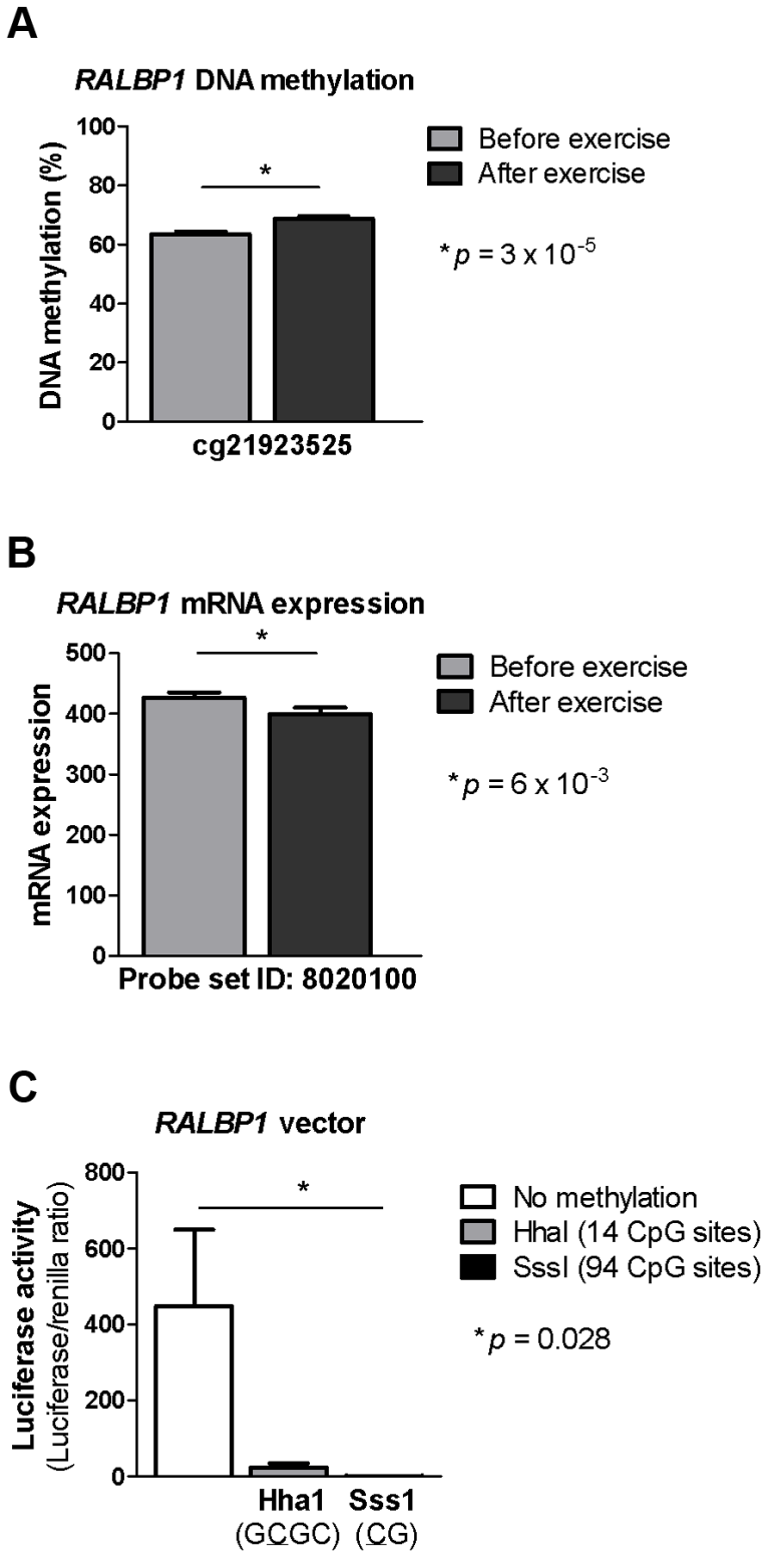
DNA methylation of RALBP1 is associated with a decrease in gene expression. A CpG site in the promoter region of *RALBP1* showed A) increased DNA methylation in response to exercise as well as B) a decrease in mRNA expression. C) *In vitro* DNA methylation of the *RALBP1* promoter decreased gene expression, as measured by luciferase activity. The result represents the mean of three independent experiments, and the values in each experiment are the mean of five replicates (background control subtracted). Data is presented as mean ± SEM.

*RALBP1* belongs to the genes that exhibit increased DNA methylation in the promoter region in parallel with decreased mRNA expression in adipose tissue in response to exercise (Figure 4a–b and Table S3). It has previously been shown to play a central role in the pathogenesis of metabolic syndrome [31] and to be involved in insulin-stimulated Glut4 trafficking [32]. We proceeded to functionally test if increased DNA methylation of the promoter of *RALBP1* may cause decreased gene expression using a reporter gene construct in which 1500 bp of DNA of the human *RALBP1* promoter was inserted into a luciferase expression plasmid that completely lacks CpG dinucleotides. The reporter construct could thereby be used to study the effect of promoter DNA methylation on the transcriptional activity. The construct was methylated using two different methyltransferases; SssI and HhaI, which methylate all CpG sites or only the internal cytosine residue in a GCGC sequence, respectively.

Increased DNA methylation of the *RALBP1* promoter, as measured by luciferase activity, suppressed the transcriptional activity of the promoter (*p* = 0.028, Figure 4c). When the *RALBP1* reporter construct was methylated *in vitro* using SssI (CG, 94 CpG sites), the transcriptional activity was almost completely disrupted (1.4±0.5), whereas the HhaI enzyme (GCGC, methylating 14 CpG sites) suppressed the transcriptional activity to a lesser extent (23.4±11.6), compared with the transcriptional activity of the mock-methylated control construct (448.2±201.7; Figure 4c).

### DNA methylation of obesity and type 2 diabetes candidate genes in human adipose tissue

We proceeded to investigate if candidate genes for obesity or T2D, identified using genome-wide association studies [3], are found among the genes exhibiting changed levels of DNA methylation in adipose tissue in response to six months exercise. Among all 476,753 CpG sites analyzed on the Infinium HumanMethylation450 BeadChip and passing QC, 1,351 sites mapped to 53 genes suggested to contribute to obesity in the review by McCarthy, and 1,315 sites mapped to 39 genes suggested to contribute to T2D [3]. We found 24 CpG sites located within 18 of the candidate genes for obesity with a difference in DNA methylation in adipose tissue in response to the exercise intervention (*q*<0.05, Table 4). Additionally, two of those genes (*CPEB4* and *SDCCAG8*) showed concurrent inverse change in mRNA expression after exercise (*q*<0.05). Among the T2D candidate genes, 45 CpG sites in 21 different genes were differentially methylated (*q*<0.05) in adipose tissue before vs. after exercise (Table 5). Of note, 10 of these CpG sites mapped to *KCNQ1* and 6 sites mapped to *TCF7L2*. A simultaneous change in mRNA expression was seen for four of the T2D candidate genes (*HHEX*, *IGF2BP2*, *JAZF1* and *TCF7L2*) where mRNA expression decreased while DNA methylation increased in response to exercise (*q*<0.05, Table 5).

**Table 4:** Individual CpG sites located within/near candidate genes for obesity [3], with a significant change in DNA methylation in adipose tissue in response to exercise.

*Obesity candidate genes*			*DNA methylation (%)*						*mRNA expression*				
Probe ID	Nearest Gene	Location	Before exercise	After exercise	Diffe-rence	*p*-value	*q*-value	Cross-reactive probes	Before exercise	After exercise	Diffe-rence	*p*-value	*q*-value
cg05501868	*ADAMTS9*	Body	62.3±4.1	66.5±4.5	4.1	6×10^−4^	0.039		218.5±46.5	219.7±59.8	1.3	>0.05	
cg07233933	*CPEB4*	Body	76.9±2.1	79.2±1.7	2.3	1×10^−4^	0.013		377.9±63.9	319.3±51.1	−58.7	<1×10^−5^	0.002
cg09141413	*GPRC5B*	TSS200; CpG Island	15.9±2.4	13.7±1.8	−2.2	6×10^−4^	0.039		476.3±69.3	448.5±58.4	−27.8	>0.05	
cg22380033	*GRB14*	1stExon; 5′UTR; CpG Island	2.1±0.4	2.6±0.4	0.5	3×10^−4^	0.029		65.1±11.4	63.0±16.8	−2.1	>0.05	
cg07645296	*ITPR2*	TSS1500; S Shore	56.2±4.5	60.1±4.6	3.9	1×10^−4^	0.013		421.5±63.1	401.6±91.0	−19.9	>0.05	
cg13203394	*ITPR2*	Body	56.8±4.4	63.3±3.2	6.5	5×10^−7^	0.001		421.5±63.1	401.6±91.0	−19.9	>0.05	
cg02212836	*LY86*	1stExon	40.0±2.4	42.7±2.5	2.7	9×10^−5^	0.013		44.2±17.0	57.8±41.0	13.6	>0.05	
cg05021589	*LY86*	TSS200	39.7±2.9	43.5±3.0	3.7	3×10^−5^	0.013		44.2±17.0	57.8±41.0	13.6	>0.05	
cg09249494	*LY86*	TSS200	31.4±3.9	35.2±3.4	3.9	1×10^−4^	0.013		44.2±17.0	57.8±41.0	13.6	>0.05	
cg16681597	*LYPLAL1*	Body; CpG Island	9.6±1.6	11.1±2.1	1.6	5×10^−4^	0.038		146.7±34.7	165.0±28.7	18.3	0.019	0.08
cg01362115	*MAP2K5*	Body	76.3±2.4	78.2±2.1	2.0	7×10^−4^	0.043		205.2±23.8	195.0±24.6	−10.2	>0.05	
cg02328326	*MAP2K5*	Body	79.6±5.8	84.8±3.8	5.2	6×10^−4^	0.043		205.2±23.8	195.0±24.6	−10.2	>0.05	
cg20055861	*MAP2K5*	Body	67.4±3.9	71.9±2.9	4.5	1×10^−4^	0.013		205.2±23.8	195.0±24.6	−10.2	>0.05	
cg27519910	*MSRA*	Body; S Shelf	76.0±2.3	78.6±2.3	2.7	4×10^−5^	0.013	48	120.4±12.5	124.0±13.5	3.6	>0.05	
cg20147645	*MTIF3*	5′UTR; N Shore	60.0±3.3	64.1±3.6	4.0	3×10^−4^	0.024		331.3±42.3	331.7±39.8	0.4	>0.05	
cg16420308	*NRXN3*	Body; N Shore	87.3±2.4	89.5±1.7	2.3	7×10^−4^	0.043		34.4±4.1	34.7±5.4	0.3	>0.05	
cg16592301	*PRKD1*	Body	84.0±2.7	86.3±2.4	2.3	8×10^−4^	0.048		182.1±20.5	177.4±28.0	−4.7	>0.05	
cg16104450	*SDCCAG8*	Body; N Shore	40.5±3.9	44.2±3.6	3.7	1×10^−4^	0.013		258.8±22.1	234.9±32.9	−23.9	1×10^−3^	0.008
cg08222913	*STAB1*	Body; CpG Island	63.2±5.1	67.8±4.2	4.6	2×10^−5^	0.013		224.1±56.3	214.6±44.2	−9.6	>0.05	
cg26104752	*TBX15*	5′UTR; S Shore	5.8±1.4	6.8±1.5	1.0	7×10^−5^	0.013		374.1±37.2	368.1±50.9	−6.0	>0.05	
cg19694781	*TMEM160*	Body; N Shore	56.1±6.5	58.3±7.0	2.3	1×10^−4^	0.013	48	205.0±25.6	231.1±25.7	26.1	1×10^−3^	0.008
cg05003666	*TUB*	TSS200;Body;CpG Island	21.8±2.5	18.4±2.3	−3.4	3×10^−4^	0.029		73.6±6.7	72.7±8.0	−0.9	>0.05	
cg01610165	*ZNF608*	Body	10.5±2.3	13.1±2.3	2.6	7×10^−4^	0.043		171.2±22.9	162.7±25.1	−8.5	>0.05	
cg12817840	*ZNF608*	Body	21.8±2.5	25.8±3.8	3.9	9×10^−5^	0.013		171.2±22.9	162.7±25.1	−8.5	>0.05	

Data are presented as mean ± SD, based on paired non-parametric test (DNA methylation) or t-test (mRNA expression) and two-tailed *p*-values. Cross-reactive probes: Maximum number of bases (≥47) matched to cross-reactive target as reported by Chen et al. [27].

**Table 5:** Individual CpG sites located within/near candidate genes for T2D [3], with a significant change in DNA methylation in adipose tissue in response to exercise.

*Type 2 diabetes candidate genes*			*DNA methylation (%)*						*mRNA expression*				
Probe ID	Nearest Gene	Location	Before exercise	After exercise	Diffe-rence	*p*-value	*q*-value	Cross-reactive probes	Before exercise	After exercise	Diffe-rence	*p*-value	*q*-value
cg05501868	*ADAMTS9*	Body	62.3±4.1	66.5±4.5	4.1	6×10^−4^	0.025		218.5±46.5	219.7±59.8	1.3	>0.05	
cg21527616	*ADAMTS9*	Body	63.6±4.7	67.0±3.3	3.4	1×10^−3^	0.044		218.5±46.5	219.7±59.8	1.3	>0.05	
cg14567877	*ADCY5*	Body	80.7±4.3	84.2±3.8	3.5	2×10^−4^	0.015		257.2±48.3	253.1±44.8	−4.1	>0.05	
cg03720898	*ARAP1*	Body	73.7±3.7	77.1±2.3	3.4	9×10^−5^	0.013	48	209.4±35.0	202.8±36.4	−6.6	>0.05	
cg06838038	*ARAP1*	Body	42.4±3.9	46.1±3.6	3.7	4×10^−5^	0.011		209.4±35.0	202.8±36.4	−6.6	>0.05	
cg10495997	*ARAP1*	5′UTR; S Shore	61.8±3.2	64.0±2.9	2.2	2×10^−4^	0.015		209.4±35.0	202.8±36.4	−6.6	>0.05	
cg15279866	*ARAP1*	5′UTR; Body	56.4±3.5	58.8±3.9	2.4	1×10^−3^	0.042		209.4±35.0	202.8±36.4	−6.6	>0.05	
cg27058763	*ARAP1*	Body; S Shelf	57.1±4.0	60.7±3.3	3.5	1×10^−3^	0.041		209.4±35.0	202.8±36.4	−6.6	>0.05	
cg01865786	*BCL11A*	Body	64.7±4.3	67.7±2.8	3.0	8×10^−4^	0.034		19.4±2.1	21.3±2.7	1.8	0.009	0.04
cg03390300	*CDKAL1*	Body	85.2±2.3	87.5±1.8	2.4	2×10^−5^	0.011		263.1±24.7	268.3±25.1	5.2	>0.05	
cg07562918	*CDKN2A*	1stExon; CpG Island	16.7±2.1	18.4±2.2	1.6	1×10^−3^	0.042		35.9±5.3	40.5±7.0	4.6	0.023	0.09
cg20836993	*DGKB*	Body	68.3±1.8	70.0±1.7	1.7	6×10^−4^	0.025	48	16.8±3.5	17.8±3.9	0.9	>0.05	
cg01602287	*DUSP8*	Body; CpG Island	75.4±4.5	79.5±3.4	4.2	1×10^−3^	0.042		97.7±13.9	94.9±13.5	−2.9	>0.05	
cg26902557	*DUSP8*	Body	49.1±3.9	52.1±4.0	3.0	6×10^−4^	0.028		97.7±13.9	94.9±13.5	−2.9	>0.05	
cg26580413	*FTO*	Body	61.0±4.4	64.3±3.8	3.3	1×10^−3^	0.044		785.0±80.7	794.4±64.7	9.4	>0.05	
cg20180364	*HHEX*	TSS1500; N Shore	46.8±4.1	50.4±3.5	3.6	2×10^−4^	0.015		172.7±24.7	144.9±30.3	−27.8	5×10^−5^	<0.001
cg16965605	*HMGA2*	Body	70.2±5.6	75.1±4.3	4.9	1×10^−3^	0.037		32.2±3.2	34.9±3.6	2.7	0.004	0.025
cg17182048	*HMGA2*	Body	81.2±3.9	84.5±4.8	3.4	1×10^−3^	0.044		32.2±3.2	34.9±3.6	2.7	0.004	0.025
cg17518348	*HMGA2*	Body	78.8±3.7	83.3±3.5	4.5	2×10^−4^	0.015		32.2±3.2	34.9±3.6	2.7	0.004	0.025
cg06150454	*IGF2BP2*	Body	54.6±4.2	58.4±2.8	3.8	3×10^−4^	0.021	48	105.6±16.5	88.4±14.9	−17.1	<1×10^−5^	<0.001
cg13918631	*IGF2BP2*	Body	66.8±4.7	70.7±3.8	3.9	2×10^−4^	0.015		105.6±16.5	88.4±14.9	−17.1	<1×10^−5^	<0.001
cg02963803	*JAZF1*	Body	59.6±2.6	62.1±2.5	2.5	9×10^−5^	0.013	48	238.2±26.1	218.5±28.0	−19.7	0.01	0.047
cg01689159	*KCNQ1*	Body; CpG Island	80.5±2.6	83.3±1.7	2.7	3×10^−4^	0.017		67.0±7.0	66.1±7.4	−0.9	>0.05	
cg03660952	*KCNQ1*	Body	51.8±3.5	55.0±2.6	3.2	1×10^−5^	0.011		67.0±7.0	66.1±7.4	−0.9	>0.05	
cg04894537	*KCNQ1*	Body	40.5±3.5	44.6±4.3	4.2	3×10^−4^	0.021		67.0±7.0	66.1±7.4	−0.9	>0.05	
cg06838584	*KCNQ1*	Body	46.8±3.7	44.0±3.4	−2.7	2×10^−4^	0.015		67.0±7.0	66.1±7.4	−0.9	>0.05	
cg08160246	*KCNQ1*	Body	60.3±3.3	63.5±3.3	3.2	5×10^−4^	0.025		67.0±7.0	66.1±7.4	−0.9	>0.05	
cg13577072	*KCNQ1*	Body	67.4±3.1	71.8±3.5	4.5	6×10^−5^	0.011		67.0±7.0	66.1±7.4	−0.9	>0.05	
cg15910264	*KCNQ1*	Body	81.4±2.8	84.3±1.9	2.9	3×10^−5^	0.011		67.0±7.0	66.1±7.4	−0.9	>0.05	
cg19672982	*KCNQ1*	Body	70.4±3.0	73.3±2.8	2.9	1×10^−4^	0.014		67.0±7.0	66.1±7.4	−0.9	>0.05	
cg24725201	*KCNQ1*	Body	91.9±1.6	93.4±1.4	1.5	7×10^−4^	0.031		67.0±7.0	66.1±7.4	−0.9	>0.05	
cg25786675	*KCNQ1*	Body	66.3±3.7	62.4±3.6	−3.9	6×10^−5^	0.011		67.0±7.0	66.1±7.4	−0.9	>0.05	
cg04775232	*PRC1*	Body	82.1±2.4	84.0±2.2	1.9	2×10^−3^	0.048		64.3±12.0	59.8±15.8	−4.5	>0.05	
cg01902845	*PROX1*	Body	73.5±5.0	77.7±3.4	4.2	6×10^−4^	0.025		21.2±6.2	21.2±7.0	0	>0.05	
cg14545834	*PTPRD*	Body; CpG Island	68.0±2.8	71.2±2.2	3.2	2×10^−4^	0.015	49	80.3±17.8	81.8±14.5	1.4	>0.05	
cg00831931	*TCF7L2*	Body	82.4±2.7	84.8±2.5	2.4	2×10^−4^	0.015		529.9±58.9	474.0±74.7	−55.8	0.001	0.008
cg05923857	*TCF7L2*	Body	72.6±5.2	76.4±3.8	3.8	8×10^−4^	0.034		529.9±58.9	474.0±74.7	−55.8	0.001	0.008
cg06403317	*TCF7L2*	Body	92.1±2.6	94.2±1.7	2.1	1×10^−3^	0.037		529.9±58.9	474.0±74.7	−55.8	0.001	0.008
cg09022607	*TCF7L2*	Body; S Shore	25.5±4.5	21.2±3.1	−4.3	6×10^−4^	0.025		529.9±58.9	474.0±74.7	−55.8	0.001	0.008
cg19226647	*TCF7L2*	1stExon; N Shore	4.4±1.0	5.5±1.3	1.1	4×10^−5^	0.011		529.9±58.9	474.0±74.7	−55.8	0.001	0.008
cg23951816	*TCF7L2*	Body	63.8±3.8	68.4±3.5	4.6	5×10^−4^	0.025		529.9±58.9	474.0±74.7	−55.8	0.001	0.008
cg01649611	*THADA*	Body	38.5±5.3	42.5±4.6	4.0	2×10^−4^	0.015		285.2±29.9	291.7±31.4	6.5	>0.05	
cg12277798	*THADA*	Body; S Shelf	77.2±4.4	81.6±3.3	4.5	5×10^−4^	0.025		285.2±29.9	291.7±31.4	6.5	>0.05	
cg16417416	*WFS1*	Body	63.9±3.5	66.9±2.9	2.9	1×10^−3^	0.044		132.2±21.6	123.6±14.5	−8.6	0.036	0.13
cg22051204	*ZBED3*	5′UTR; S Shore	51.1±3.5	53.5±3.2	2.4	1×10^−3^	0.042		187.9±22.9	189.1±17.4	1.3	>0.05	

Data are presented as mean ± SD, based on paired non-parametric test (DNA methylation) or t-test (mRNA expression) and two-tailed *p*-values. Cross-reactive probes: Maximum number of bases (≥47) matched to cross-reactive target as reported by Chen et al. [27].

### Silencing of *Hdac4* and *Ncor2* in 3T3-L1 adipocytes is associated with increased lipogenesis

**Figure 5:**
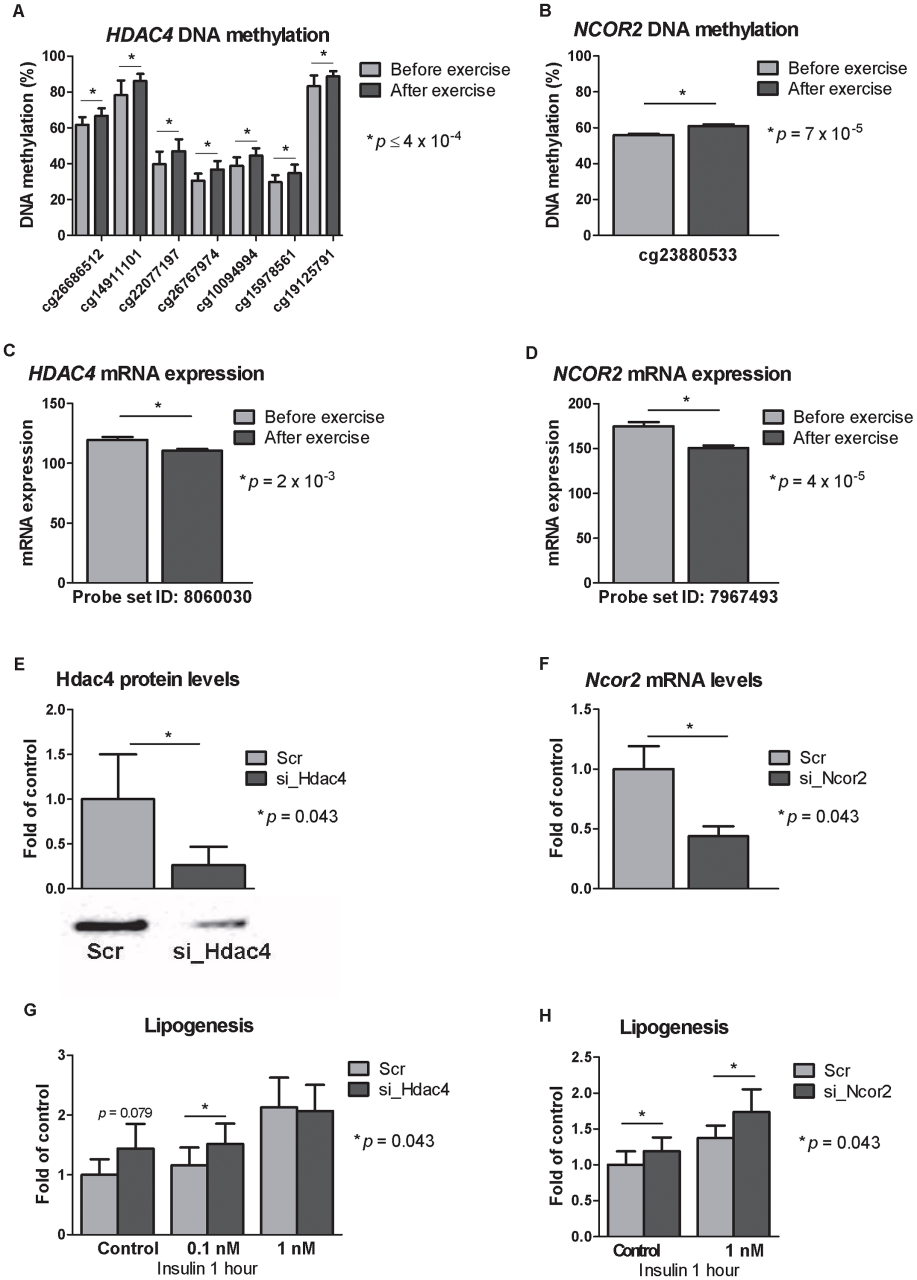
Silencing of Hdac4 and Ncor2 in 3T3-L1 adipocytes results in increased lipogenesis. CpG sites in the promoter region of A) *HDAC4* and B) *NCOR2* showed increased DNA methylation in response to exercise as well as decreased mRNA expression (C–D). Knock-downs were verified either by E) Western blot analysis (for Hdac4) or F) by qRT-PCR (for *Ncor2*). Lipogenesis increased in 3T3-L1 adipocytes where G) *Hdac4* (*n* = 5) or H) *Ncor2* (*n* = 5) had been silenced. Data is presented as mean ± SEM.

To further understand if the genes that exhibit differential DNA methylation and mRNA expression in adipose tissue *in vivo* affect adipocyte metabolism, we silenced the expression of selected genes in 3T3-L1 adipocytes using siRNA and studied its effect on lipogenesis. Two of the genes where we found increased DNA methylation in parallel with decreased mRNA expression in human adipose tissue in response to exercise (Figure 5a–d and Table S3) were selected for functional studies in a 3T3-L1 adipocyte cell line. *HDAC4* was further a strong candidate due to multiple affected CpG sites within the gene, and both *HDAC4* and *NCOR2* are biologically interesting candidates in adipose tissue and the pathogenesis of obesity and type 2 diabetes [33]–[35]. Silencing of *Hdac4* and *Ncor2* in the 3T3-L1 adipocytes resulted in 74% reduction in the Hdac4 protein level (1.00±0.50 vs. 0.26±0.20, *p* = 0.043; Figure 5e) while the *Ncor2* mRNA level was reduced by 56% (1.00±0.19 vs. 0.44±0.08, *p* = 0.043; Figure 5f) of control after transfection with siRNA for 72 hours and 24 h, respectively. Lipogenesis was nominally increased in the basal state (1.00±0.26 vs. 1.44±0.42, *p* = 0.079) and significantly increased in response to 0.1 nM insulin (1.16±0.30 vs. 1.52±0.34, *p* = 0.043) in 3T3-L1 adipocytes with decreased Hdac4 levels (Figure 5g). Decreased Ncor2 levels also resulted in increased lipogenesis in the basal (1.00±0.19 vs. 1.19±0.19, *p* = 0.043) and insulin stimulated (1 nM; 1.38±0.17 vs. 1.73±0.32, *p* = 0.043) state (Figure 5h).

### Technical validation of Infinium HumanMethylation450 BeadChip DNA methylation data

To technically validate the DNA methylation data from the Infinium HumanMethylation450 BeadChips, we compared the genome-wide DNA methylation data from one adipose tissue sample analyzed at four different occasions. Technical reproducibility was observed between all samples, with Pearson's correlation coefficients >0.99 (*p*<2.2×10^−16^, Figure S1a). Secondly, we re-analyzed DNA methylation of four CpG sites using Pyrosequencing (PyroMark Q96ID, Qiagen) in adipose tissue of all 23 men both before and after exercise (Table S4). We observed a significant correlation between the two methods for each CpG site (*p*<0.05; Figure S1b), and combining all data points gives a correlation factor of 0.77 between the two methods (*p*<0.0001; Figure S1c).

## Discussion

This study highlights the dynamic feature of DNA methylation, described using a genome-wide analysis in human adipose tissue before and after exercise. We show a general global increase in adipose tissue DNA methylation in response to 6 months exercise, but also changes on the level of individual CpG sites, with significant absolute differences ranging from 0.2–10.9%. This data, generated using human adipose tissue biopsies, demonstrate an important role for epigenetic changes in human metabolic processes. Additionally, this study provides a first reference for the DNA methylome in adipose tissue from healthy, middle aged men.

Changes in DNA methylation have been suggested to be a biological mechanism behind the beneficial effects of physical activity [18], [36]. In line with this theory, a nominal association between physical activity level and global LINE-1 methylation in leukocytes was recently reported [37]. More important from a metabolic point-of-view, a study investigating the impact of long term exercise intervention on genome-wide DNA methylation in human skeletal muscle was recently published, and showed epigenetic alterations of genes important for T2D pathogenesis and muscle physiology [23]. This relationship between exercise and altered DNA methylation is here expanded to include human adipose tissue, as our data show 17,975 individual CpG sites that exhibit differential DNA methylation in adipose tissue after an exercise intervention, corresponding to 7,663 unique genes throughout the genome. Genome-wide association studies have identified multiple SNPs strongly associated with disease, but still the effect sizes of the common variants influencing for example risk of T2D are modest and in total only explain a small proportion of the predisposition. Importantly, although each variant only contributes with a small risk, these findings have led to improved understanding of the biological basis of disease [3]. Similarly, the absolute changes in DNA methylation observed in response to the exercise intervention are modest, but the large number of affected sites may in combination potentially contribute to a physiological response. Moreover, if the exercise induced differences in DNA methylation is expressed as fold-change instead of absolute differences, we observe changes ranging from 6 to 38%.

In regard to the distribution of analyzed CpG sites, most of the differentially methylated sites were found within the gene bodies and in intergenic regions, and fewer than expected was found in the promoter regions and CpG islands. This is in agreement with previous studies showing that differential DNA methylation is often found in regions other than CpG islands. For example, it was shown that tissue-specific differentially methylated regions in the 5′UTR are strongly underrepresented within CpG islands [38] and that most tissue-specific DNA methylation occurs at CpG island shores rather than the within CpG islands, and also in regions more distant than 2 kb from CpG islands [39]. It has further been proposed that non-CpG island DNA methylation is more dynamic than methylation within CpG islands [40]. The importance of differential DNA methylation within gene bodies is supported by multiple studies showing a positive correlation between gene body methylation and active transcription [40], and that DNA methylation may regulate exon splicing [41], [42]. In this study, the exercise intervention associated with a decrease in waist circumference and waist-hip ratio, which suggests reduced abdominal obesity, a phenotype known to be associated with reduced risk of metabolic diseases [43]. Indeed, increased levels of DNA methylation were observed after exercise both in the promoter region and in the gene body of *ITPR2*, a locus previously associated with waist-hip ratio [44]. Furthermore, in addition to increased VO_2max_, the study participants responded to exercise with a decrease in diastolic blood pressure and heart rate, and an improvement in HDL levels, which are some of the different mechanisms through which exercise is known to reduce the risk for T2D and cardiovascular disease [43]. Adipose tissue comprises not only of adipocytes but a mixture of different cell types. To evaluate if the cellular composition of adipose tissue may change during exercise, we looked at the mRNA expression for a number of cell type specific markers before and after the exercise intervention. None of these showed any difference in adipose tissue mRNA expression before vs. after exercise (*q*>0.05; *LEP*, *PNPLA2*, *FAS*, *LIPE* and *PPARG* as markers of adipocytes; *SEBPA/B/D* and *DLK1* as markers of preadipocytes, *PRDM16* and *UCP1* as markers of brown adipocytes; *ITGAX*, *EMR1*, *ITGAM* as markers of macrophages; *TNF* and *IL6* representing cytokines and finally *CCL2* and *CASP7* as markers for inflammation). Although this result suggests that there is no a major change in the cellular composition of the adipose tissue studied before compared with after the exercise intervention, future studies should investigate the methylome in isolated adipocytes. Additionally, in previous studies of DNA methylation in human pancreatic islets, the differences observed in the mixed-cell tissue were also detected in clonal beta cells exposed to hyperglycemia [20], [21], suggesting that in at least some tissues, the effects are transferable from the relevant cell type to the tissue of interest for human biology.

The impact of this study is further strengthened by our results showing altered DNA methylation of genes or loci previously associated with obesity and T2D. Although there was no enrichment of differential DNA methylation in those genes compared to the whole dataset, this result may provide a link to the mechanisms for how the loci associated with common diseases exert their functions [18]. 18 obesity and 21 T2D candidate genes had one or more CpG sites which significantly changed in adipose tissue DNA methylation after exercise. 10 CpG sites were found to have altered DNA methylation in response to exercise within the gene body of *KCNQ1*, a gene encoding a potassium channel and known to be involved in the pathogenesis of T2D, and also subject to parental imprinting [45]. Moreover, exercise associated with changes in DNA methylation of six intragenic CpG sites in *TCF7L2*, the T2D candidate gene harbouring a common variant with the greatest described effect on the risk of T2D [3]. This is of particular interest considering that *TCF7L2* is subject to alternative splicing [46], [47] and the fact that gene exons are more highly methylated than introns, with DNA methylation spikes at splice junctions, suggesting a possible role for differential DNA methylation in transcript splicing [42]. In addition to differential DNA methylation, we also observed an inverse change in adipose tissue mRNA expression for some of these candidate genes, including *TCF7L2*, *HHEX*, *IGF2BP2*, *JAZF1*, *CPEB4* and *SDCCAG8* in response to exercise.

The understanding of the human methylome is incomplete although recently developed methods for genome-wide analysis of DNA methylation already have made, and are likely to continue to make, tremendous advances [48]. High coverage data describing differences in the levels of DNA methylation between certain human tissues or cell types [38], as well as differences observed during development [42], have started to emerge. Regardless, deeper knowledge about the epigenetic architecture and regulation in human adipose tissue has been missing until now. We found that the genetic region with the highest average level of DNA methylation in adipose tissue was the 3′UTR, followed by the gene body and intergenic regions, and those regions also increased the level of DNA methylation in response to exercise. This supports the view that the human methylome can dynamically respond to changes in the environment [14], [15]. One explanation for the low average levels of DNA methylation observed in the promoter region (TSS1500/200), 5′UTR and the first exon, may be that these regions often overlap with CpG islands, which are generally known to be unmethylated. Indeed, our results show a very low level of DNA methylation within the CpG islands, and how the level then increases with increasing distances to a CpG island.

It has long been debated if increased DNA methylation precedes gene silencing, or if it is rather a consequence of altered gene activity [40]. The luciferase assay experiments from this study and others [21], [23] suggest that DNA methylation may have a causal role, as increased promoter DNA methylation leads to reduced transcriptional activity. Here we further related our findings of altered DNA methylation to mRNA expression, and we identified 197 genes where both DNA methylation and mRNA expression significantly changed in adipose tissue after exercise. Of these, 115 genes (58%) showed an inverse relation, 97% showing an increase in the level of DNA methylation and a decrease in mRNA expression. It should be noted that epigenetic processes are likely to influence more aspects of gene expression, including accessibility of the gene, posttranscriptional RNA processing and stability, splicing and also translation [49]. For example, DNA methylation within the gene body has previously been linked to active gene transcription, suggestively by improving transcription efficiency [42].

Two genes, *HDAC4* and *NCOR2*, with biological relevance in adipose tissue metabolism were selected for functional validation. HDAC4 is a histone deacetylase regulated by phosphorylation, and known to repress GLUT4 transcription in adipocytes [35]. In skeletal muscle, HDAC4 has been found to be exported from the nucleus during exercise, suggesting that removal of the transcriptional repressive function could be a mechanism for exercise adaptation [50]. For *HDAC4*, we observed increased levels of DNA methylation and a simultaneous decrease in mRNA expression in adipose tissue in response to the exercise intervention. Additionally, the functional experiments in cultured adipocytes suggested increased lipogenesis when *Hdac4* expression was reduced. This could be an indicator of reduced repressive activity on GLUT4, leading to an increase in adipocyte glucose uptake and subsequent incorporation of glucose into triglycerides in the process of lipogenesis. *NCOR2* also exhibited increased levels of DNA methylation and a simultaneous decrease in mRNA expression in adipose tissue in response to the exercise intervention, and furthermore we observed increased lipogenesis when *Ncor2* expression was down regulated in the 3T3-L1 cell line. NCOR2 is a nuclear co-repressor, involved in the regulation of genes important for adipogenesis and lipid metabolism, and with the ability to recruit different histone deacetylase enzymes, including HDAC4 [51]. These results may be of clinical importance, since HDAC inhibitors have been suggested in the treatment of obesity and T2D [18], [52].

In summary, this study provides a detailed map of the human methylome in adipose tissue, which can be used as a reference for further studies. We have also found evidence for an association between differential DNA methylation and mRNA expression in response to exercise, as well as a connection to genes known to be involved in the pathogenesis of obesity and T2D. Finally, functional validation in adipocytes links DNA methylation via gene expression to altered metabolism, supporting the role of histone deacetylase enzymes as a potential candidate in clinical interventions.

## Materials and Methods

### Ethics statement

Written informed consent was obtained from all participants and the research protocol was approved by the local human research ethics committee.

### Study participants

This study included a total of 31 men from Malmö, Sweden, recruited for a six months exercise intervention study, as previously described [23], [53]. Fifteen of the individuals had a first-degree family history of T2D (FH^+^), whereas sixteen individuals had no family history of diabetes (FH^−^). They were all sedentary, but healthy, with a mean age of 37.4 years and a mean BMI of 27.8 kg/m^2^ at inclusion. All subjects underwent a physical examination, an oral glucose tolerance test and a submaximal exercise stress test. Bioimpedance was determined to estimate fat mass with a BIA 101 Body Impedance Analyzer (Akern Srl, Pontassieve, Italy). To directly assess the maximal oxygen uptake (VO_2max_), an ergometer bicycle (Ergomedic 828E, Monark, Sweden) was used together with heart rate monitoration (Polar T61, POLAR, Finland) [53]. FH^+^ and FH^−^ men were group-wise matched for age, BMI and physical fitness (VO_2max_) at baseline. Subcutaneous biopsies of adipose tissue from the right thigh were obtained during the fasting state under local anaesthesia (1% Lidocaine) using a 6 mm Bergström needle (Stille AB, Sweden) from all participants before and from 23 participants after the six months exercise intervention (>48 hours after the last exercise session). The weekly group training program included one session of 1 hour spinning and two sessions of 1 hour aerobics and was led by a certified instructor. The participation level was on average 42.8±4.5 sessions, which equals to 1.8 sessions/week of this endurance exercise intervention. The study participants were requested to not change their diet and daily activity level during the intervention.

### Genome-wide DNA methylation analysis

DNA methylation was analyzed in DNA extracted from adipose tissue, using the Infinium HumanMethylation450 BeadChip assay (Illumina, San Diego, CA, USA). This array contains 485,577 probes, which cover 21,231 (99%) RefSeq genes [25], [54]. Genomic DNA (500 ng) from adipose tissue was bisulfite treated using the EZ DNA methylation kit (Zymo Research, Orange, CA, USA). Analysis of DNA methylation with the Infinium assay was carried out on the total amount of bisulfite-converted DNA, with all other procedures following the standard Infinium HD Assay Methylation Protocol Guide (Part #15019519, Illumina). The BeadChips' images were captured using the Illumina iScan. The raw methylation score for each probe represented as methylation β-values was calculated using GenomeStudio Methylation module software (β = intensity of the Methylated allele (M)/intensity of the Unmethylated allele (U)+intensity of the Methylated allele (M)+100). All included samples showed a high quality bisulfite conversion efficiency (intensity signal >4000) [55], and also passed all GenomeStudio quality control steps based on built in control probes for staining, hybridization, extension and specificity. Individual probes were then filtered based on Illumina detection *p*-value and all CpG sites with a mean *p*<0.01 were considered detected and used for subsequent analysis. In total we obtained DNA methylation data for 476,753 CpG sites from adipose tissue of 31 men before and 23 men after the exercise intervention. Before further analysis, the DNA methylation data was exported from GenomeStudio and subsequently analyzed using Bioconductor [56] and the lumi package [57]. β-values were converted to M-values (M = log^2^(β/(1-β))), a more statistically valid method for conducting differential methylation analysis [58]. Next, data was background corrected by subtracting the median M-value of the 600 built in negative controls and was further normalized using quantile normalization. Correction for batch effects within the methylation array data was performed using COMBAT [59]. For the calculations of global DNA methylation, quantile normalization was omitted and probes reported to be cross-reactive (≥49 bases) or directly affected by a SNP (MAF>5%) were removed [27]. Due to different performance of Infinium I and Infinium II assays [25], the results based on average DNA methylation are calculated and presented separately for each probe type. To control for technical variability within the experiment, one adipose tissue sample was included and run on four different occasions (Figure S1a). As the β-value is easier to interpret biologically, M-values were reconverted to β-values when describing the results and creating the figures.

### mRNA expression analysis

RNA extracted from the subcutaneous adipose tissue biopsies was used for a microarray analysis, performed using the GeneChip Human Gene 1.0 ST whole transcript based array (Affymetrix, Santa Clara, CA, USA), following the Affymetrix standard protocol. Basic Affymetrix chip and experimental quality analyses were performed using the Expression Console Software, and the robust multi-array average method (RMA) was used for background correction, data normalization and probe summarization [60].

### Luciferase assay

The human promoter fragment containing 1500 bp of DNA upstream of the transcription start site for *RALBP1* (Chr18:9474030–9475529, GRCh37/hg19) was inserted into a CpG-free luciferase reporter vector (pCpGL-basic) as previously described [21]. The construct was methylated using two different DNA methyltransferases; SssI which methylates all cytosine residues within the double-stranded dinucleotide recognition sequence CG, and HhaI which methylates only the internal cytosine residue in the GCGC sequence (New England Biolabs, Frankfurt, Germany). INS-1 cells were co-transfected with 100 ng of the pCpGL-vector without (control) or with any of the three *RALBP1* inserts (no DNA methyltransferase, SssI, HhaI) together with 2 ng of pRL renilla luciferase control reporter vector as a control for transfection efficiency (Promega, Madison, WI, USA). Firefly luciferase activity, as a value of expression, was measured for each construct and normalized against renilla luciferase activity using the TD-20/20 luminometer (Turner Designs, Sunnyvale, CA, USA). The results represent the mean of three independent experiments, and the values in each experiment are the mean of five replicates. Cells transfected with an empty pCpGL-vector were used as background control in each experiment.

### siRNA transfection of 3T3-L1 adipocytes and lipogenesis assay

For detailed description of siRNA and lipogenesis experiments see Methods S1. Briefly, 3T3-L1 fibroblasts were cultured at sub-confluence in DMEM containing 10% (v/v) FCS, 100 U/ml penicillin and 100 µg/ml streptomycin at 37°C and 95% air/5% CO_2_. Two-day post-confluent cells were incubated for 72 h in DMEM supplemented with 0.5 mM IBMX, 10 µg/ml insulin and 1 µM dexamethasone, after which the cells were cultured in normal growth medium. Seven days post-differentiation, cells were transfected by electroporation with 2 nmol of each siRNA sequence/gene (Table S5). 0.2 nmol scrambled siRNA of each low GC-, medium GC- and high GC-complex were mixed as control. The cells were replated after transfection and incubated for 72 hours (siRNA against *Hdac4*) or 24 hours (siRNA against *Ncor2*).

Cells harvested for western blot analysis were solubilized and homogenized, and 20 µg protein was subjected to SDS-PAGE (4–12% gradient) and subsequent transferred to nitrocellulose membranes. The primary antibody (rabbit polyclonal anti-hdac4; ab12172, Abcam, Cambridge, UK) was diluted in 5 ml 5% BSA/TBST and incubated overnight in 4°C. The secondary antibody (goat anti-rabbit IgG conjugated to horseradish peroxidase; ALI4404, BioSource, Life Technologies Ltd, Paisley, UK) was diluted 1∶20,000 in 5% milk/TBST. Protein was detected using Super Signal and ChemiDoc (BioRad, Hercules, CA, USA).

Quantitative PCR (Q-PCR) analyses were performed in triplicate on an ABI7900 using Assays on demand with TaqMan technology (Mm00448796_m1, Applied Biosystems, Carlsbad, CA, USA). The mRNA expression was normalized to the expression of the endogenous control gene *Hprt* (Mm01545399_m1, Applied Biosystems).

To measure lipogenesis, 10 µl tritium labelled ([^3^H]) glucose (Perkin Elmer, Waltham, MA, USA) was added followed by insulin of different concentrations; 0, 0.1, and 1 nM for *Hdac4* siRNA and 0 and 1 nM for *Ncor2* siRNA experiments, respectively. All concentrations were tested in duplicates. After 1 hour, incorporation of [^3^H] glucose into cellular lipids was measured by scintillation counting. Lipogenesis is expressed as fold of basal lipogenesis.

### DNA methylation analysis using PyroSequencing

PyroSequencing (PyroMark Q96ID, Qiagen, Hilden, Germany) was used to technically validate data from the genome-wide DNA methylation analysis. PCR and sequencing primers were either designed using PyroMark Assay Design 2.0 or ordered as pre-designed methylation assays (Qiagen, Table S4), and all procedures were performed according to recommended protocols. Briefly, 100 ng genomic DNA from adipose tissue of 23 individuals both before and after the exercise intervention was bisulfite converted using Qiagen's EpiTect kit. With one primer biotinylated at its 5′ end, bisulfite-converted DNA was amplified by PCR using the PyroMark PCR Master Mix kit (Qiagen). Biotinylated PCR products were immobilized onto streptavidin-coated beads (GE Healthcare, Uppsala, Sweden) and DNA strands were separated using denaturation buffer. After washing and neutralizing using PyroMark Q96 Vacuum Workstation, the sequencing primer was annealed to the immobilized strand. PyroSequencing was performed with the PyroMark Gold Q96 reagents and data were analyzed using the PyroMark Q96 (version 2.5.8) software (Qiagen).

### Statistical analysis

Clinical data is presented as mean ± SD, and comparisons based on a t-test and two-tailed *p*-values. Genome-wide DNA methylation data from the Infinium HumanMethylation450 BeadChip before vs. after the six month exercise intervention was analyzed using a paired non-parametric test, whereas a paired t-test was used to compare the mRNA expression. DNA methylation and mRNA expression data are expressed as mean ± SD. To account for multiple testing and reduce the number of false positives, we applied *q*-values to measure the false discovery rate (FDR) on our genome-wide analyses of DNA methylation and mRNA expression [24]. Luciferase activity was analyzed using the Friedman test (paired, non-parametric test on dependent samples) and presented as mean ± SEM. Data from 3T3-L1 adipocyte experiments showing protein, mRNA and lipogenesis levels are presented as mean ± SEM, and the results are based on Wilcoxon signed-rank test.

## Supporting Information

Figure S1 Technical validation. A) Technical replicate of one adipose tissue DNA sample included in the study, analyzed using the Infinium HumanMethylation450 BeadChip on four different occasions. B–C) Data obtained from all adipose tissue samples for four CpG sites, from both the Infinium HumanMethylation450 BeadChip (x axis) and using Pyrosequencing (y axis).
(TIF)

Methods S1 Detailed descriptions of small interfering RNA transfection, mRNA expression analysis, lipogenesis assay and statistical analysis.
(DOC)

Table S1 Baseline clinical characteristics of individuals with (FH^+^) or without (FH^−^) a family history of type 2 diabetes.
(DOC)

Table S2 Average DNA methylation for regions in relation to nearest gene or CpG islands, separately for Infinium I and II assays, respectively.
(DOC)

Table S3 CpG sites with a change in DNA methylation (*q*<0.05 and difference in β≥5%) concurrent with an inverse change in mRNA expression (*q*<0.05) of the nearest gene, in response to the exercise intervention study.
(DOC)

Table S4 Assay design for technical validation of DNA methylation data using PyroSequencing.
(DOC)

Table S5 siRNA assays.
(DOC)
