# Supplementary figures and images for: A Six Months Exercise Intervention Influences the Genome-wide DNA Methylation Pattern in Human Adipose Tissue
Source: PLoS Genet. 2013 Jun 27;9(6):e1003572. doi: 10.1371/journal.pgen.1003572 (PMC3694844; doi:10.1371/journal.pgen.1003572)

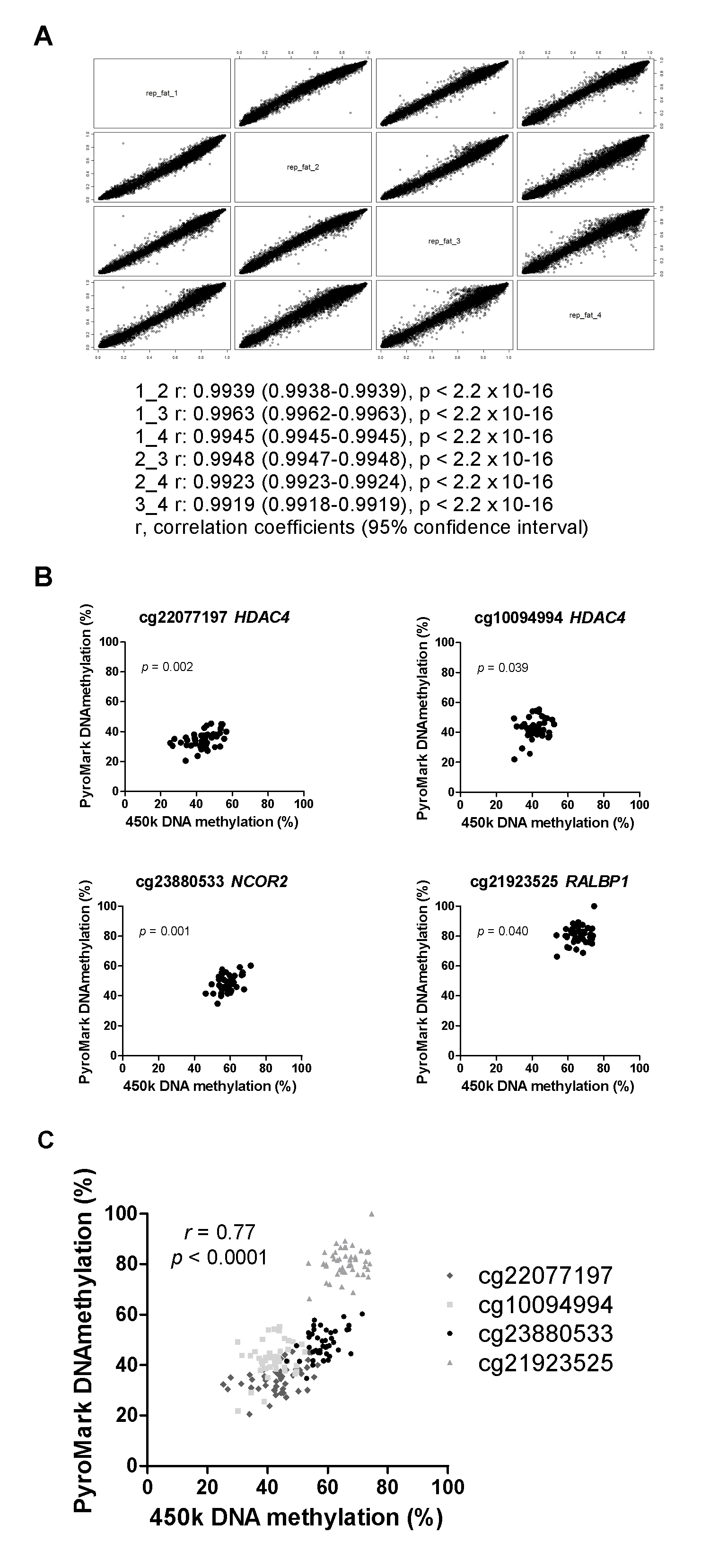

Supplement: Figure S1 — Technical validation. A) Technical replicate of one adipose tissue DNA sample included in the study, analyzed using the Infinium HumanMethylation450 BeadChip on four different occasions. B–C) Data obtained from all adipose tissue samples for four CpG sites, from both the Infinium HumanMethylation450 BeadChip (x axis) and using Pyrosequencing (y axis). (TIF) [file pgen.1003572.s001.tif]
