# Supplementary material for: A Six Months Exercise Intervention Influences the Genome-wide DNA Methylation Pattern in Human Adipose Tissue
Source: PLoS Genet. 2013 Jun 27;9(6):e1003572. doi: 10.1371/journal.pgen.1003572 (PMC3694844; doi:10.1371/journal.pgen.1003572)
Supplement: Methods S1 — Detailed descriptions of small interfering RNA transfection, mRNA expression analysis, lipogenesis assay and statistical analysis. (DOC) [file pgen.1003572.s002.doc]

**Methods S1**

## Small interfering RNA transfection

Seven days post differentiation, cells were transfected by electroporation. The cells were rinsed twice with PBS. 1 ml trypsin/EDTA (0.05%/0.02% weight/volume) and 0.5 ml sterile filtered collagenase type 1 (5mg/ml in PBS) were used to dissociate and disaggregate the cells. The cells were collected in complete DMEM, rinsed in PBS twice and resuspended in PBS. 400 µl cell suspension and 2 nmol of each siRNA sequence/gene (Table S2) were mixed in a 0.4 cm cuvette. 0.2 nmol scrambled siRNA of each low GC-, medium GC- and high GC-complex were mixed as control. The cells were electroporated at 0.18 kV and 960 µFarad. Directly afterwards cells were rescued in complete DMEM and rested for about 10 minutes. The cells were re-plated to 12-well plates and incubated for 24 hours (siRNA against Ncor2) or for 72 hours (siRNA against Hdac4). Cells from two 10 cm dishes were used to one 12-well plate.

Cells harvested for western blot analysis were lysed in homogenization buffer containing 50 mM Tris-HCl pH 7.5, 1 mM EGTA, 1 mM EDTA, 1% w/v NP40, 1 mM Na-orthovanadate, 50 mM NaF, 5 mM Na-pyrophosphate and 0.27 M sucrose, including protease inhibitors, 10 μg/ml antipain, 10 μg/ml leupeptin and 1 μg/ml pepstatin A. Cells harvested for Q-PCR were solubilized in 200 µl PBS. The cell suspensions were collected and stored at -20°C for western blot analysis and -80°C for Q-PCR.

## mRNA expression analysis

RNA from the 3T3-L1 adipocytes was extracted using RNeasy minikit (cat no. 74104, Qiagen). Cells were homogenized using a 0.60 mm, 23G syringe. RNA concentrations were measured using NanoDrop (ND-1000, Saveen & Werner, Sweden). 750 ng RNA was converted to cDNA using QuantiTect Reverse Transcription kit (Qiagen). cDNA was diluted six times and Q-PCR was run in triplicates on an ABI 7900 using Assays on demand (Applied Biosystems, Carlsbad, CA, USA; Table S2). The mRNA expression was normalized to the expression of the endogenous control, *Hprt* (Applied Biosystems; Table S2) and the relative gene expression levels were calculated from the differences in Ct-values (ΔΔC_t_).

## Lipogenesis assay

To measure lipogenesis, cells were rinsed with PBS twice and incubated for two hours in KRH buffer-low glucose (Krebs-Ringer medium, 25mM HEPES, 3.5% w/v) bovine serum albumin (BSA) and 2 mM glucose (pH 7.4). 10 µl tritium labelled ([^3^H]) glucose (45.7 Ci/mmol, 1mCi/ml, Perkin Elmer, Waltham, MA, USA) diluted 1:45 was added followed by insulin of different concentrations; 0, 0.1 and 1 nM for Hdac4 siRNA and 0 and 1 nM for Ncor2 siRNA experiments, respectively. After a 1 hour incubation, cells were collected in 2.5 ml scintillation liquid (0.3 g/l 1,4-bis[5-phenyl-2-oxazolyl]benzene, 2,2′-p-phenylene-bis[5-phenyloxazole] (POPOP) and 5 g/l 2,5-diphenyl oxazole (PPO)). Incorporation of [^3^H] glucose into cellular lipids was measured by scintillation counting.

## Statistical analysis

All data showing protein, mRNA and lipogenesis levels are presented as mean ± standard error of the mean (SEM). Wilcoxon signed-rank test was used to evaluate results.
