# Supplementary material for: A Six Months Exercise Intervention Influences the Genome-wide DNA Methylation Pattern in Human Adipose Tissue
Source: PLoS Genet. 2013 Jun 27;9(6):e1003572. doi: 10.1371/journal.pgen.1003572 (PMC3694844; doi:10.1371/journal.pgen.1003572)
Supplement: Table S1 — Baseline clinical characteristics of individuals with (FH+) or without (FH−) a family history of type 2 diabetes. (DOC) [file pgen.1003572.s003.doc]

**Table S1.** Baseline clinical characteristics of individuals with (FH^+^) or without (FH^-^) a family history of type 2 diabetes.

| ***Characteristics*** | ***FH^+^*** *(n=15)* | ***FH^-^*** *(n=16)* | ***P-value*** |
| --- | --- | --- | --- |
| Age (years) | 38.7 ± 4.4 | 36.2 ± 3.9 | 0.10 |
| BMI (kg/m^2^) | 27.6 ± 2.8 | 28.1 ± 3.0 | 0.63 |
| Fasting glucose (mmol/L) | 4.31 ± 0.39 | 4.36 ± 0.60 | 0.79 |
| HbA1c (%) | 4.39 ± 0.35 | 4.25 ± 0.26 | 0.23 |
| VO_2max_ (mL/kg/min) | 33.6 ± 5.0 | 31.4 ± 4.4 | 0.21 |

Data are expressed as mean ± SD, based on two-sample t-tests and two-tailed *p*-values.
