# Supplementary material for: A Six Months Exercise Intervention Influences the Genome-wide DNA Methylation Pattern in Human Adipose Tissue
Source: PLoS Genet. 2013 Jun 27;9(6):e1003572. doi: 10.1371/journal.pgen.1003572 (PMC3694844; doi:10.1371/journal.pgen.1003572)
Supplement: Table S2 — Average DNA methylation for regions in relation to nearest gene or CpG islands, separately for Infinium I and II assays, respectively. (DOC) [file pgen.1003572.s004.doc]

**Table S2**. Average DNA methylation for regions in relation to nearest gene or CpG islands, separately for Infinium I and II assays, respectively.

| **Region in relation to**  **Nearest Gene** | **Infinium I Assays DNA Methylation (%)** | | | | | **Infinium II Assays DNA Methylation (%)** | | | |
| --- | --- | --- | --- | --- | --- | --- | --- | --- | --- |
|  | ***n*** | **Before Exercise** | **After Exercise** | | ***q*-value** | ***n*** | **Before Exercise** | **After Exercise** | ***q*-value** |
| TSS1500 | 21,649 | 10.13 ± 0.56 | 9.67 ± 0.70 | | 6x10^-5^ | 59,155 | 35.02 ± 1.63 | 35.82 ± 1.59 | 3x10^-3^ |
| TSS200 | 29,917 | 6.12 ± 0.47 | 5.77 ± 0.54 | | 6x10^-5^ | 30,900 | 21.24 ± 1.22 | 21.55 ± 1.03 | 0.06 |
| 5'UTR | 22,561 | 8.39 ± 0.52 | 8.01 ± 0.64 | | 9x10^-5^ | 40,497 | 33.59 ± 1.57 | 34.39 ± 1.54 | 3x10^-3^ |
| 1stExon | 17,675 | 6.47 ± 0.46 | 6.16 ± 0.57 | | 9x10^-5^ | 20,394 | 23.23 ± 1.28 | 23.68 ± 1.10 | 8x10^-3^ |
| Body | 39,899 | 50.74 ± 1.01 | 51.33 ± 0.84 | | 0.06 | 124,403 | 60.27 ± 1.98 | 61.75 ± 2.13 | 8x10^-4^ |
| 3'UTR | 3,053 | 64.37 ± 1.37 | 65.46 ± 1.25 | | 8x10^-3^ | 15,256 | 69.56 ± 2.07 | 71.28 ± 2.22 | 8x10^-4^ |
| Intergenic | 23,993 | 43.15 ± 1.12 | 43.40 ± 0.95 | | 0.26 | 82,670 | 58.23 ± 2.04 | 59.60 ± 2.12 | 2x10^-3^ |
| **Region in relation to**  **CpG Islands** | **Infinium I Assays DNA Methylation (%)** | | | | | **Infinium II Assays DNA Methylation (%)** | | | |
|  | ***n*** | **Before Exercise** | **After Exercise** | ***q*-value** | | ***n*** | **Before Exercise** | **After Exercise** | ***q*-value** |
| N Shelf | 3,322 | 74.41 ± 1.27 | 75.54 ± 1.24 | 2x10^-3^ | | 19,536 | 70.69 ± 2.01 | 72.28 ± 2.10 | 5x10^-4^ |
| N Shore | 11,370 | 31.83 ± 0.66 | 31.52 ± 0.72 | 0.12 | | 48,323 | 42.47 ± 1.80 | 43.41 ± 1.79 | 2x10^-3^ |
| Island | 74,887 | 9.84 ± 0.57 | 9.45 ± 0.70 | 5x10^-4^ | | 69,979 | 19.90 ± 1.14 | 20.27 ± 1.02 | 0.01 |
| S Shore | 9,710 | 34.52 ± 0.67 | 34.27 ± 0.69 | 0.20 | | 37,005 | 40.35 ± 1.76 | 41.22 ± 1.72 | 2x10^-3^ |
| S Shelf | 2,902 | 74.71 ± 1.33 | 75.88 ± 1.27 | 2x10^-3^ | | 17,468 | 71.38 ± 2.03 | 72.96 ± 2.09 | 5x10^-4^ |
| Open Sea | 24,613 | 69.41 ± 1.34 | 70.44 ± 1.27 | 8x10^-3^ | | 134,329 | 67.37 ± 2.01 | 68.84 ± 2.12 | 7x10^-4^ |

*n*, number of probes. Data are presented as mean ± SD.
