# Supplementary material for: A Six Months Exercise Intervention Influences the Genome-wide DNA Methylation Pattern in Human Adipose Tissue
Source: PLoS Genet. 2013 Jun 27;9(6):e1003572. doi: 10.1371/journal.pgen.1003572 (PMC3694844; doi:10.1371/journal.pgen.1003572)
Supplement: Table S3 — CpG sites with a change in DNA methylation (q<0.05 and difference in β≥5%) concurrent with an inverse change in mRNA expression (q<0.05) of the nearest gene, in response to the exercise intervention study. (DOC) [file pgen.1003572.s005.doc]

**Table S3.** CpG sites with a change in DNA methylation (*q*<0.05 and difference in β≥5%) concurrent with an inverse change in mRNA expression (*q*<0.05) of the nearest gene, in response to the exercise intervention study.

| **Nearest Gene** | **Chr** | ***DNA Methylation (%)*** | | | | | | | ***mRNA Expression*** | | | | | |
| --- | --- | --- | --- | --- | --- | --- | --- | --- | --- | --- | --- | --- | --- | --- |
|  |  | **Probe ID** | **Location** | **Before Exercise** | **After Exercise** | **Change** | ***p*-value** | ***q*-value** | **Probe set ID** | **Before Exercise** | **After Exercise** | **Change** | ***p*-value** | ***q*-value** |
| *ABR* | 17 | cg01881265 | Body;CpG Island | 53 ± 3 | 58 ± 4 | 5 | 3x10^-6^ | 0.01 | 8010983 | 225.6±34.6 | 199.5±30.6 | -26,2 | 2x10^-4^ | 1x10^-3^ |
| *ABR* | 17 | cg26682335 | Body | 61 ± 9 | 70 ± 7 | 9 | 3x10^-4^ | 0.02 | 8010983 | 225.6±34.6 | 199.5±30.6 | -26,2 | 2x10^-4^ | 1x10^-3^ |
| *AGAP1* | 2 | cg11905061 | Body;S Shore | 38 ± 4 | 43 ± 4 | 5 | 3x10^-5^ | 0.01 | 8049448 | 225.8±37.3 | 188.8±22.2 | -37,0 | 6x10^-5^ | 4x10^-4^ |
| *AGAP1* | 2 | cg27634020 | Body | 58 ± 6 | 63 ± 4 | 5 | 3x10^-4^ | 0.02 | 8049448 | 225.8±37.3 | 188.8±22.2 | -37,0 | 6x10^-5^ | 4x10^-4^ |
| *AGGF1* | 5 | cg15817406 | TSS1500;N Shore | 52 ± 4 | 57 ± 3 | 5 | 3x10^-5^ | 0.01 | 8106429 | 368.6±27.3 | 337.6±47.3 | -31,0 | 1x10^-3^ | 3x10^-3^ |
| *AHI1* | 6 | cg18685455 | Body | 45 ± 5 | 50 ± 5 | 5 | 8x10^-4^ | 0.04 | 8129728 | 242.5±35.9 | 212±46.6 | -30,5 | 2x10^-3^ | 0.01 |
| *ANKFY1* | 17 | cg23942064 | Body | 69 ± 6 | 76 ± 6 | 7 | 2x10^-4^ | 0.02 | 8011599 | 261.8±27.9 | 246.1±21.1 | -15,6 | 7x10^-3^ | 0.01 |
| *ATAD2B* | 2 | cg10208083 | Body;N Shelf | 77 ± 5 | 82 ± 4 | 5 | 9x10^-5^ | 0.01 | 8050658 | 185.9±21 | 167.3±25.4 | -18,6 | 4x10^-3^ | 0.01 |
| *ATP11A* | 13 | cg02417360 | Body | 70 ± 6 | 77 ± 5 | 7 | 5x10^-4^ | 0.03 | 7970162 | 152.1±22 | 139.5±20.5 | -12,6 | 2x10^-2^ | 0.03 |
| *ATP11A* | 13 | cg26800525 | Body;S Shore | 72 ± 6 | 78 ± 5 | 6 | 2x10^-5^ | 0.01 | 7970162 | 152.1±22 | 139.5±20.5 | -12,6 | 2x10^-2^ | 0.03 |
| *BCL2* | 18 | cg08223235 | Body | 61 ± 6 | 66 ± 4 | 5 | 3x10^-4^ | 0.03 | 8023646 | 383.7±54.1 | 351.6±45.5 | -32,1 | 4x10^-3^ | 0.01 |
| *BCL2* | 18 | cg23756272 | Body | 42 ± 5 | 47 ± 5 | 5 | 3x10^-4^ | 0.02 | 8023646 | 383.7±54.1 | 351.6±45.5 | -32,1 | 4x10^-3^ | 0.01 |
| *BCL2* | 18 | cg25059899 | Body | 55 ± 5 | 61 ± 4 | 6 | 3x10^-5^ | 0.01 | 8023646 | 383.7±54.1 | 351.6±45.5 | -32,1 | 4x10^-3^ | 0.01 |
| *BCORL1* | X | cg11304846 | Body | 57 ± 6 | 63 ± 6 | 6 | 7x10^-4^ | 0.03 | 8169882 | 108.1±15.4 | 98.7±10 | -9,4 | 5x10^-3^ | 0.01 |
| *BTNL9* | 5 | cg22319784 | 3'UTR;CpG Island | 29 ± 5 | 34 ± 5 | 5 | 9x10^-5^ | 0.01 | 8110631 | 825.1±188.6 | 683.7±199.7 | -141,3 | 1x10^-3^ | 3x10^-3^ |
| *C1QTNF7* | 4 | cg14547240 | TSS1500;Body;5'UTR | 59 ± 6 | 52 ± 3 | -7 | 5x10^-4^ | 0.03 | 8094184 | 191.1±74.5 | 239.1±105.7 | 48,0 | 9x10^-3^ | 0.02 |
| *CCDC88C* | 14 | cg13027206 | Body | 67 ± 6 | 72 ± 5 | 5 | 5x10^-4^ | 0.03 | 7980828 | 124.9±32.5 | 100.4±28 | -24,5 | 2x10^-3^ | 0.01 |
| *CHD8* | 14 | cg12774429 | 3'UTR;TSS1500;S Shore | 74 ± 6 | 79 ± 3 | 5 | 1x10^-4^ | 0.02 | 7977693 | 233.3±15.6 | 214.7±21.5 | -18,6 | 5x10^-4^ | 2x10^-3^ |
| *CRTC3* | 15 | cg22396498 | Body | 60 ± 5 | 65 ± 5 | 5 | 1x10^-6^ | 4x10^-3^ | 7986049 | 219.7±29.5 | 196.2±26.2 | -23,5 | 9x10^-4^ | 3x10^-3^ |
| *CTNNA1* | 5 | cg07894651 | 5'UTR | 76 ± 6 | 82 ± 4 | 6 | 3x10^-4^ | 0.02 | 8108378 | 1320±84.3 | 1239.5±111.9 | -80,5 | 1x10^-4^ | 7x10^-4^ |
| *CUGBP1* | 11 | cg13308137 | 5'UTR | 59 ± 5 | 65 ± 5 | 6 | 2x10^-5^ | 0.01 | 7947894 | 487.4±36.8 | 464.9±40.9 | -22,5 | 2x10^-2^ | 0.03 |
| *CUGBP2* | 10 | cg04221117 | Body;TSS1500;N Shore | 53 ± 6 | 59 ± 4 | 6 | 2x10^-5^ | 0.01 | 7926127 | 512.3±42.4 | 468.6±39.9 | -43,8 | 7x10^-5^ | 5x10^-4^ |
| *DGKD* | 2 | cg13053135 | Body | 66 ± 6 | 72 ± 5 | 6 | 3x10^-4^ | 0.02 | 8049317 | 138.9±16.1 | 123.6±12.7 | -15,3 | 4x10^-4^ | 2x10^-3^ |
| *DLC1* | 8 | cg08768218 | TSS200 | 46 ± 5 | 51 ± 5 | 5 | 2x10^-4^ | 0.02 | 8149413 | 466.7±54.6 | 439.8±58.4 | -26,9 | 3x10^-2^ | 0.04 |
| *DLC1* | 8 | cg10349685 | TSS200 | 32 ± 4 | 38 ± 5 | 6 | 2x10^-5^ | 0.01 | 8149413 | 466.7±54.6 | 439.8±58.4 | -26,9 | 3x10^-2^ | 0.04 |
| *ECEL1* | 2 | cg01856162 | Body;CpG Island | 22 ± 6 | 16 ± 5 | -6 | 6x10^-4^ | 0.03 | 8059748 | 54±8.3 | 58.6±5.6 | 4,6 | 9x10^-3^ | 0.02 |
| *EHMT1* | 9 | cg14495033 | Body;N Shore | 56 ± 9 | 63 ± 6 | 7 | 3x10^-5^ | 0.01 | 8159702 | 205.1±18.2 | 183.4±15.8 | -21,8 | 1x10^-5^ | 1x10^-4^ |
| *EHMT2* | 6 | cg14246190 | Body;N Shelf | 65 ± 4 | 70 ± 3 | 5 | 2x10^-6^ | 5x10^-3^ | 8125172 | 133.5±13.1 | 124.2±9.9 | -9,4 | 2x10^-3^ | 0.01 |
| *EIF2S2* | 20 | cg18589543 | Body;N Shelf | 59 ± 5 | 64 ± 4 | 5 | 5x10^-5^ | 0.01 | 8065730 | 298.5±29.8 | 272.7±58.3 | -25,8 | 7x10^-3^ | 0.01 |
| *EIF4EBP2* | 10 | cg24877964 | TSS1500;N Shore | 43 ± 6 | 50 ± 7 | 7 | 4x10^-4^ | 0.03 | 7928119 | 2513.1±375.9 | 2345±340.9 | -168,1 | 3x10^-2^ | 0.04 |
| *ERICH1* | 8 | cg03761569 | Body;N Shore | 83 ± 7 | 89 ± 4 | 6 | 6x10^-4^ | 0.03 | 8148985 | 317±47.4 | 258.4±41.3 | -58,5 | 4x10^-7^ | 2x10^-5^ |
| *EVL* | 14 | cg01485838 | Body | 61 ± 7 | 68 ± 5 | 7 | 1x10^-5^ | 0.01 | 7976726 | 145.6±18 | 130.8±17.4 | -14,8 | 3x10^-3^ | 0.01 |
| *FBXL20* | 17 | cg25661397 | Body | 63 ± 5 | 68 ± 5 | 5 | 4x10^-4^ | 0.03 | 8014825 | 207.1±25.9 | 193.3±20.7 | -13,8 | 3x10^-2^ | 0.04 |
| *FIGNL2* | 12 | cg07375978 | 1stExon;CpG Island | 52 ± 5 | 57 ± 5 | 5 | 2x10^-3^ | 0.05 | 7963328 | 36.8±5.4 | 32.9±4.3 | -3,9 | 9x10^-3^ | 0.02 |
| *FIGNL2* | 12 | cg19856499 | 1stExon;CpG Island | 43 ± 4 | 48 ± 5 | 5 | 5x10^-4^ | 0.03 | 7963328 | 36.8±5.4 | 32.9±4.3 | -3,9 | 9x10^-3^ | 0.02 |
| *FOXK1* | 7 | cg26136772 | Body;N Shelf | 67 ± 4 | 73 ± 5 | 6 | 2x10^-3^ | 0.05 | 8131253 | 214.3±28.5 | 181.3±21.8 | -33,0 | 1x10^-5^ | 2x10^-4^ |
| *FOXP1* | 3 | cg22828884 | Body | 63 ± 4 | 71 ± 4 | 8 | 2x10^-5^ | 0.01 | 8088776 | 250.2±28.2 | 234.3±32.6 | -15,9 | 1x10^-2^ | 0.02 |
| *GABBR1* | 6 | cg23801674 | Body | 54 ± 8 | 61 ± 7 | 7 | 1x10^-3^ | 0.04 | 8178298 | 187±46.7 | 151±24.4 | -36,0 | 3x10^-4^ | 1x10^-3^ |
| *GABBR1* | 6 | cg23801674 | Body | 54 ± 8 | 61 ± 7 | 7 | 1x10^-3^ | 0.04 | 8124654 | 162.3±35.6 | 135.5±21 | -26,8 | 5x10^-4^ | 2x10^-3^ |
| *GABBR1* | 6 | cg23801674 | Body | 54 ± 8 | 61 ± 7 | 7 | 1x10^-3^ | 0.04 | 8179595 | 187.3±47.7 | 149.4±26 | -37,8 | 1x10^-4^ | 7x10^-4^ |
| *GAK* | 4 | cg14140403 | Body;CpG Island | 54 ± 5 | 60 ± 5 | 6 | 3x10^-4^ | 0.02 | 8098789 | 200.6±17.8 | 187.2±19.1 | -13,3 | 1x10^-2^ | 0.02 |
| *GAS8* | 16 | cg07378013 | TSS1500;N Shore | 67 ± 5 | 74 ± 4 | 7 | 1x10^-4^ | 0.02 | 7998103 | 175.9±23.8 | 160.2±18.4 | -15,6 | 5x10^-3^ | 0.01 |
| *GATA2* | 3 | cg00241663 | 5'UTR;CpG Island | 43 ± 5 | 49 ± 5 | 6 | 3x10^-6^ | 0.01 | 8090469 | 99.5±10.8 | 91.8±11.9 | -7,7 | 5x10^-3^ | 0.01 |
| *GNPNAT1* | 14 | cg18214930 | 5'UTR;N Shelf | 70 ± 6 | 76 ± 4 | 6 | 4x10^-4^ | 0.03 | 7979196 | 191.7±29 | 171.4±24.6 | -20,3 | 8x10^-3^ | 0.02 |
| *GSN* | 9 | cg14186336 | 5'UTR | 52 ± 5 | 57 ± 5 | 5 | 2x10^-4^ | 0.02 | 8157582 | 5066.1±467 | 4741.4±402 | -324,7 | 2x10^-2^ | 0.03 |
| *GSPT2* | X | cg02239640 | 1stExon;S Shore | 45 ± 7 | 50 ± 5 | 5 | 3x10^-4^ | 0.02 | 8167654 | 132±10.4 | 125.6±14.6 | -6,4 | 2x10^-2^ | 0.03 |
| *HCFC1* | X | cg05707218 | Body;N Shore | 34 ± 6 | 40 ± 5 | 6 | 2x10^-4^ | 0.02 | 8175947 | 126.8±18.5 | 111.5±13.9 | -15,3 | 2x10^-4^ | 1x10^-3^ |
| *HDAC4* | 2 | cg10094994 | Body | 39 ± 5 | 45 ± 4 | 6 | 3x10^-5^ | 0.01 | 8060030 | 119.7±12.3 | 110.4±9.5 | -9,3 | 2x10^-3^ | 5x10^-3^ |
| *HDAC4* | 2 | cg14911101 | Body;CpG Island | 78 ± 8 | 86 ± 4 | 8 | 2x10^-4^ | 0.02 | 8060030 | 119.7±12.3 | 110.4±9.5 | -9,3 | 2x10^-3^ | 5x10^-3^ |
| *HDAC4* | 2 | cg15978561 | Body;CpG Island | 30 ± 4 | 35 ± 5 | 5 | 2x10^-5^ | 0.01 | 8060030 | 119.7±12.3 | 110.4±9.5 | -9,3 | 2x10^-3^ | 5x10^-3^ |
| *HDAC4* | 2 | cg19125791 | Body | 83 ± 6 | 89 ± 3 | 6 | 4x10^-4^ | 0.03 | 8060030 | 119.7±12.3 | 110.4±9.5 | -9,3 | 2x10^-3^ | 5x10^-3^ |
| *HDAC4* | 2 | cg22077197 | Body | 40 ± 7 | 47 ± 7 | 7 | 3x10^-4^ | 0.03 | 8060030 | 119.7±12.3 | 110.4±9.5 | -9,3 | 2x10^-3^ | 5x10^-3^ |
| *HDAC4* | 2 | cg26686512 | Body;S Shore | 62 ± 4 | 67 ± 4 | 5 | 2x10^-5^ | 0.01 | 8060030 | 119.7±12.3 | 110.4±9.5 | -9,3 | 2x10^-3^ | 5x10^-3^ |
| *HDAC4* | 2 | cg26767974 | Body | 31 ± 4 | 37 ± 5 | 6 | 5x10^-6^ | 0.01 | 8060030 | 119.7±12.3 | 110.4±9.5 | -9,3 | 2x10^-3^ | 5x10^-3^ |
| *HOXA9* | 7 | cg03217995 | Body;N Shore | 51 ± 5 | 58 ± 5 | 7 | 1x10^-5^ | 0.01 | 8138749 | 131.1±17.7 | 120.5±20.3 | -10,6 | 1x10^-2^ | 0.02 |
| *HOXA9* | 7 | cg21007852 | Body;N Shore | 50 ± 5 | 55 ± 5 | 5 | 7x10^-5^ | 0.01 | 8138749 | 131.1±17.7 | 120.5±20.3 | -10,6 | 1x10^-2^ | 0.02 |
| *HSPG2* | 1 | cg26112909 | 3'UTR;Body;S Shore | 60 ± 5 | 65 ± 5 | 5 | 3x10^-5^ | 0.01 | 7913450 | 892.7±183.8 | 740.1±105 | -152,5 | 3x10^-4^ | 1x10^-3^ |
| *INPP4A* | 2 | cg22526076 | 5'UTR | 64 ± 5 | 71 ± 3 | 7 | 7x10^-5^ | 0.01 | 8043791 | 230.5±23.1 | 213±15.6 | -17,5 | 1x10^-3^ | 3x10^-3^ |
| *KIAA0182* | 16 | cg08899895 | Body;S Shore | 62 ± 3 | 67 ± 3 | 5 | 1x10^-5^ | 0.01 | 7997680 | 490.7±50.6 | 449.9±52.1 | -40,7 | 1x10^-3^ | 4x10^-3^ |
| *KTN1* | 4 | cg03926751 | 5'UTR;N Shore | 48 ± 5 | 53 ± 5 | 5 | 6x10^-4^ | 0.03 | 7974483 | 799.5±109.3 | 625.5±173.2 | -174,0 | 4x10^-6^ | 6x10^-5^ |
| *KTN1* | 14 | cg23685650 | TSS1500;Body;N Shore | 49 ± 5 | 54 ± 5 | 5 | 2x10^-4^ | 0.02 | 7974483 | 799.5±109.3 | 625.5±173.2 | -174,0 | 4x10^-6^ | 6x10^-5^ |
| *LATS2* | 13 | cg20902277 | Body;S Shore | 75 ± 6 | 80 ± 5 | 5 | 1x10^-3^ | 0.04 | 7970498 | 282±15.9 | 258.6±18.4 | -23,4 | 1x10^-5^ | 1x10^-4^ |
| *LIMD1* | 3 | cg13148151 | Body;S Shelf | 64 ± 5 | 69 ± 4 | 5 | 2x10^-4^ | 0.02 | 8079334 | 169.1±21.7 | 157.7±16.2 | -11,4 | 2x10^-2^ | 0.03 |
| *LIMD1* | 3 | cg22111014 | Body | 80 ± 6 | 86 ± 5 | 6 | 1x10^-3^ | 0.04 | 8079334 | 169.1±21.7 | 157.7±16.2 | -11,4 | 2x10^-2^ | 0.03 |
| *LOX* | 5 | cg15111469 | TSS1500;S Shore | 67 ± 6 | 72 ± 4 | 5 | 2x10^-3^ | 0.05 | 8113709 | 593±118.9 | 520.5±99.7 | -72,5 | 1x10^-2^ | 0.02 |
| *LRIG3* | 12 | cg12366597 | Body | 80 ± 6 | 85 ± 4 | 5 | 2x10^-3^ | 0.05 | 7964602 | 316.7±55.3 | 292.1±52.4 | -24,6 | 2x10^-2^ | 0.03 |
| *LRRFIP1* | 2 | cg26271045 | TSS1500;CpG Island | 44 ± 5 | 49 ± 5 | 5 | 2x10^-4^ | 0.02 | 8049544 | 194.2±39.8 | 169.3±41.1 | -24,9 | 1x10^-2^ | 0.02 |
| *LRRFIP1* | 2 | cg26271045 | TSS1500;CpG Island | 44 ± 5 | 49 ± 5 | 5 | 2x10^-4^ | 0.02 | 8049532 | 149.4±37.5 | 118.9±44.3 | -30,5 | 3x10^-3^ | 0.01 |
| *LRRFIP1* | 2 | cg26271045 | TSS1500;CpG Island | 44 ± 5 | 49 ± 5 | 5 | 2x10^-4^ | 0.02 | 8049534 | 135.4±24.8 | 120.9±20.4 | -14,5 | 2x10^-2^ | 0.03 |
| *LRRFIP1* | 2 | cg26271045 | TSS1500;CpG Island | 44 ± 5 | 49 ± 5 | 5 | 2x10^-4^ | 0.02 | 8049512 | 355±56.6 | 305.5±78.6 | -49,5 | 2x10^-3^ | 0.01 |
| *MACF1* | 1 | cg19913559 | Body | 66 ± 8 | 73 ± 7 | 7 | 1x10^-3^ | 0.04 | 7900235 | 899.6±89.1 | 798±100.1 | -101,6 | 1x10^-4^ | 7x10^-4^ |
| *MAK* | 6 | cg03349251 | TSS1500 | 67 ± 7 | 73 ± 4 | 6 | 2x10^-4^ | 0.02 | 8123893 | 23.7±3.3 | 21.6±2.6 | -2,0 | 1x10^-2^ | 0.02 |
| *MAPKAP1* | 9 | cg14146657 | 5'UTR;Body | 69 ± 5 | 75 ± 5 | 6 | 1x10^-3^ | 0.04 | 8164177 | 670±54 | 626.5±63.1 | -43,5 | 3x10^-3^ | 0.01 |
| *MATN2* | 8 | cg12861602 | Body | 68 ± 5 | 74 ± 5 | 6 | 2x10^-5^ | 0.01 | 8147516 | 441.5±63.3 | 400.9±65.9 | -40,7 | 3x10^-3^ | 0.01 |
| *MSI2* | 17 | cg06078469 | Body;S Shore | 43 ± 4 | 49 ± 4 | 6 | 5x10^-7^ | 4x10^-3^ | 8008682 | 106.9±14.3 | 92.4±11.7 | -14,4 | 4x10^-4^ | 2x10^-3^ |
| *MSI2* | 17 | cg07377994 | Body | 64 ± 7 | 70 ± 5 | 6 | 7x10^-4^ | 0.03 | 8008682 | 106.9±14.3 | 92.4±11.7 | -14,4 | 4x10^-4^ | 2x10^-3^ |
| *MSI2* | 17 | cg08788712 | Body | 69 ± 6 | 75 ± 4 | 6 | 5x10^-4^ | 0.03 | 8008682 | 106.9±14.3 | 92.4±11.7 | -14,4 | 4x10^-4^ | 2x10^-3^ |
| *MSI2* | 17 | cg17178761 | Body | 35 ± 5 | 40 ± 3 | 5 | 1x10^-4^ | 0.02 | 8008682 | 106.9±14.3 | 92.4±11.7 | -14,4 | 4x10^-4^ | 2x10^-3^ |
| *MSRB3* | 12 | cg23378033 | TSS1500;N Shore | 49 ± 5 | 54 ± 4 | 5 | 3x10^-4^ | 0.03 | 7956856 | 400.3±56.4 | 361.3±54.8 | -39,0 | 5x10^-3^ | 0.01 |
| *MTMR6* | 13 | cg06094043 | 3'UTR | 69 ± 5 | 75 ± 5 | 6 | 2x10^-3^ | 0.05 | 7970655 | 226.8±29.2 | 209.4±24.9 | -17,5 | 1x10^-2^ | 0.02 |
| *MUC1* | 1 | cg06216400 | Body;N Shelf | 62 ± 6 | 68 ± 4 | 6 | 1x10^-4^ | 0.02 | 7920642 | 62.9±9.9 | 57.7±8.6 | -5,2 | 3x10^-2^ | 0.04 |
| *MYADM* | 19 | cg12970155 | 5'UTR;S Shore | 61 ± 7 | 67 ± 5 | 6 | 7x10^-5^ | 0.01 | 8031047 | 690.5±137.2 | 588.2±111 | -102,3 | 3x10^-4^ | 2x10^-3^ |
| *MYO18A* | 17 | cg25743584 | TSS1500;Body;N Shelf | 69 ± 5 | 74 ± 5 | 5 | 2x10^-4^ | 0.02 | 8013860 | 166.7±23.9 | 145.8±19.9 | -20,8 | 9x10^-4^ | 3x10^-3^ |
| *NAV1* | 1 | cg01828733 | TSS200;Body;CpG Island | 41 ± 4 | 46 ± 4 | 5 | 1x10^-6^ | 4x10^-3^ | 7908694 | 424.5±67.1 | 360.8±54.6 | -63,6 | 6x10^-5^ | 4x10^-4^ |
| *NAV1* | 1 | cg05091570 | Body;CpG Island | 31 ± 4 | 37 ± 4 | 6 | 5x10^-7^ | 4x10^-3^ | 7908694 | 424.5±67.1 | 360.8±54.6 | -63,6 | 6x10^-5^ | 4x10^-4^ |
| *NCOR2* | 12 | cg23880533 | Body | 56 ± 4 | 61 ± 4 | 5 | 7x10^-5^ | 0.01 | 7967493 | 174.8±25.1 | 150.6±15.5 | -24,2 | 4x10^-5^ | 3x10^-4^ |
| *NFIA* | 1 | cg12126901 | Body | 41 ± 7 | 47 ± 5 | 6 | 1x10^-3^ | 0.04 | 7901788 | 963.4±108.9 | 899.8±87.8 | -63,5 | 2x10^-2^ | 0.03 |
| *NFIC* | 19 | cg14101485 | Body;CpG Island | 45 ± 5 | 50 ± 4 | 5 | 1x10^-4^ | 0.02 | 8024623 | 472.2±74.7 | 417.6±49.5 | -54,6 | 2x10^-3^ | 0.01 |
| *NFIC* | 19 | cg24199203 | Body;N Shore | 49 ± 6 | 54 ± 6 | 5 | 2x10^-3^ | 0.05 | 8024623 | 472.2±74.7 | 417.6±49.5 | -54,6 | 2x10^-3^ | 0.01 |
| *NFIX* | 19 | cg13338734 | Body;S Shore | 53 ± 4 | 58 ± 4 | 5 | 7x10^-5^ | 0.01 | 8026139 | 463.3±66.3 | 385.9±66 | -77,3 | 5x10^-6^ | 7x10^-5^ |
| *NOTCH4* | 6 | cg15031299 | Body | 82 ± 6 | 88 ± 3 | 6 | 3x10^-5^ | 0.01 | 8125383 | 314.8±47.8 | 286.1±58.5 | -28,6 | 3x10^-2^ | 0.04 |
| *NR5A2* | 1 | cg07926895 | Body;S Shore | 26 ± 4 | 32 ± 4 | 6 | 8x10^-6^ | 0.01 | 7908597 | 141.6±27.2 | 119.7±26.6 | -21,8 | 2x10^-3^ | 0.01 |
| *NR5A2* | 1 | cg24553673 | Body;S Shore | 33 ± 5 | 40 ± 4 | 7 | 2x10^-7^ | 4x10^-3^ | 7908597 | 141.6±27.2 | 119.7±26.6 | -21,8 | 2x10^-3^ | 0.01 |
| *NRD1* | 1 | cg21108691 | TSS1500;Body | 75 ± 6 | 81 ± 6 | 6 | 2x10^-3^ | 0.05 | 7916077 | 825.7±78.5 | 764.2±78.4 | -61,5 | 4x10^-4^ | 2x10^-3^ |
| *NUCKS1* | 1 | cg24606240 | TSS1500;S Shore | 55 ± 8 | 64 ± 6 | 9 | 7x10^-4^ | 0.03 | 7923798 | 1610.9±137.1 | 1540.7±113.4 | -70,3 | 3x10^-2^ | 0.04 |
| *PACS2* | 14 | cg00808648 | TSS1500;N Shore | 44 ± 4 | 49 ± 4 | 5 | 5x10^-7^ | 4x10^-3^ | 7977371 | 165.8±19.9 | 147.8±19.6 | -18,1 | 1x10^-3^ | 4x10^-3^ |
| *PACS2* | 14 | cg00808648 | TSS1500;N Shore | 44 ± 4 | 49 ± 4 | 5 | 5x10^-7^ | 4x10^-3^ | 7977344 | 291±45.3 | 253.5±26.5 | -37,5 | 3x10^-4^ | 1x10^-3^ |
| *PACS2* | 14 | cg16162970 | TSS1500;N Shore | 54 ± 6 | 60 ± 4 | 6 | 3x10^-4^ | 0.03 | 7977344 | 291±45.3 | 253.5±26.5 | -37,5 | 3x10^-4^ | 1x10^-3^ |
| *PACS2* | 14 | cg16162970 | TSS1500;N Shore | 54 ± 6 | 60 ± 4 | 6 | 3x10^-4^ | 0.03 | 7977371 | 165.8±19.9 | 147.8±19.6 | -18,1 | 1x10^-3^ | 4x10^-3^ |
| *PDZD2* | 5 | cg22571544 | Body | 63 ± 4 | 69 ± 3 | 6 | 2x10^-5^ | 0.01 | 8104693 | 925.6±200.6 | 795±171.4 | -130,6 | 3x10^-3^ | 0.01 |
| *PIP4K2A* | 10 | cg14215711 | Body | 47 ± 6 | 52 ± 4 | 5 | 4x10^-4^ | 0.03 | 7932530 | 330.4±71.1 | 281±52.4 | -49,4 | 3x10^-3^ | 0.01 |
| *PLCL2* | 3 | cg15602631 | Body;TSS1500 | 80 ± 5 | 85 ± 4 | 5 | 6x10^-4^ | 0.03 | 8078187 | 151.8±23.2 | 134±21.9 | -17,8 | 1x10^-3^ | 4x10^-3^ |
| *PLEC1* | 8 | cg08455275 | Body;TSS1500;CpG Island | 37 ± 7 | 42 ± 4 | 5 | 1x10^-3^ | 0.04 | 8153568 | 236±42 | 210.7±22.1 | -25,4 | 4x10^-3^ | 0.01 |
| *PPM1K* | 4 | cg00086710 | 5'UTR;N Shore | 45 ± 5 | 50 ± 5 | 5 | 1x10^-3^ | 0.04 | 8101701 | 75.9±10.9 | 68.9±7.9 | -7,0 | 5x10^-3^ | 0.01 |
| *PPP1R12A* | 12 | cg05381383 | Body;TSS1500 | 71 ± 7 | 77 ± 5 | 6 | 1x10^-3^ | 0.04 | 7965123 | 565.4±76.8 | 460.1±110.2 | -105,3 | 1x10^-4^ | 8x10^-4^ |
| *PRKAR1B* | 7 | cg26203136 | Body | 35 ± 6 | 29 ± 5 | -6 | 3x10^-4^ | 0.03 | 8137675 | 67±9.4 | 72.8±7.5 | 5,8 | 3x10^-3^ | 0.01 |
| *PRKX* | X | cg20578501 | Body;N Shelf | 67 ± 5 | 72 ± 4 | 5 | 2x10^-4^ | 0.02 | 8171182 | 147.2±14.4 | 134.5±17.2 | -12,7 | 5x10^-4^ | 2x10^-3^ |
| *RALBP1* | 18 | cg21923525 | TSS1500;N Shore | 63 ± 5 | 69 ± 4 | 6 | 3x10^-5^ | 0.01 | 8020100 | 426.6±43.4 | 399.3±57.2 | -27,3 | 6x10^-3^ | 0.01 |
| *RASA3* | 13 | cg13643184 | Body | 62 ± 4 | 67 ± 4 | 5 | 7x10^-4^ | 0.03 | 7972946 | 413.5±58.9 | 352.5±43.8 | -60,9 | 4x10^-7^ | 1x10^-5^ |
| *RFPL2* | 22 | cg13748755 | Body | 77 ± 5 | 71 ± 6 | -6 | 1x10^-3^ | 0.04 | 8075564 | 18.3±1.5 | 19.9±3.1 | 1,6 | 2x10^-2^ | 0.03 |
| *RGS6* | 14 | cg01775802 | Body | 63 ± 10 | 71 ± 11 | 8 | 1x10^-3^ | 0.04 | 7975482 | 105.2±12 | 94.3±19.3 | -10,9 | 5x10^-3^ | 0.01 |
| *RNF216* | 7 | cg20160729 | Body | 74 ± 6 | 80 ± 5 | 6 | 8x10^-4^ | 0.04 | 8137986 | 210.9±22.8 | 192±23.1 | -18,9 | 5x10^-3^ | 0.01 |
| *RNF216* | 7 | cg20702205 | Body | 44 ± 6 | 49 ± 5 | 5 | 1x10^-3^ | 0.04 | 8137986 | 210.9±22.8 | 192±23.1 | -18,9 | 5x10^-3^ | 0.01 |
| *ROCK1* | 18 | cg05835414 | Body;N Shelf | 65 ± 5 | 71 ± 6 | 6 | 2x10^-4^ | 0.02 | 8022441 | 693.2±101.2 | 558.7±150.2 | -134,4 | 8x10^-5^ | 5x10^-4^ |
| *RUSC2* | 9 | cg13395060 | Body | 69 ± 4 | 75 ± 4 | 6 | 2x10^-5^ | 0.01 | 8155048 | 226.4±29.8 | 197.8±21.7 | -28,6 | 8x10^-5^ | 5x10^-4^ |
| *SCRN1* | 7 | cg06133110 | 5'UTR;Body;N Shore | 43 ± 6 | 48 ± 5 | 5 | 1x10^-3^ | 0.04 | 8138824 | 375.9±92.5 | 326.1±77.5 | -49,8 | 1x10^-4^ | 7x10^-4^ |
| *SFRS8* | 12 | cg21080633 | Body;CpG Island | 69 ± 6 | 75 ± 5 | 6 | 1x10^-3^ | 0.04 | 7959927 | 386.8±40.1 | 348±37.8 | -38,9 | 9x10^-4^ | 3x10^-3^ |
| *SH3D19* | 4 | cg09786062 | 5'UTR;TSS1500 | 69 ± 4 | 74 ± 5 | 5 | 8x10^-4^ | 0.04 | 8103166 | 1243.3±137.2 | 1113.8±135.1 | -129,6 | 5x10^-5^ | 4x10^-4^ |
| *SH3RF3* | 2 | cg01458510 | Body | 65 ± 5 | 70 ± 5 | 5 | 2x10^-3^ | 0.05 | 8044295 | 143.7±22.1 | 128.5±18.1 | -15,2 | 2x10^-3^ | 5x10^-3^ |
| *SHANK3* | 22 | cg09635994 | 3'UTR;S Shore | 73 ± 5 | 79 ± 5 | 6 | 1x10^-3^ | 0.04 | 8074131 | 221.6±52.6 | 179.5±31.2 | -42,1 | 8x10^-4^ | 3x10^-3^ |
| *SIN3B* | 19 | cg07826747 | Body;N Shore | 70 ± 5 | 75 ± 4 | 5 | 1x10^-3^ | 0.04 | 8026610 | 212.8±27.7 | 185.3±21.3 | -27,5 | 8x10^-5^ | 5x10^-4^ |
| *SLFN13* | 17 | cg18067859 | TSS1500;N Shore | 52 ± 4 | 57 ± 5 | 5 | 9x10^-5^ | 0.01 | 8014248 | 69±17 | 58.2±10.7 | -10,8 | 4x10^-3^ | 0.01 |
| *SLMAP* | 3 | cg18712231 | Body | 76 ± 5 | 82 ± 4 | 6 | 4x10^-5^ | 0.01 | 8080685 | 382.1±57.2 | 312.9±55.2 | -69,1 | 2x10^-4^ | 9x10^-4^ |
| *SMARCA4* | 19 | cg08151828 | 5'UTR;TSS1500 | 70 ± 6 | 78 ± 6 | 8 | 5x10^-4^ | 0.03 | 8025788 | 182±17.4 | 167.1±15.3 | -14,9 | 2x10^-3^ | 0.01 |
| *SMURF1* | 7 | cg04878240 | Body | 76 ± 5 | 82 ± 5 | 6 | 3x10^-4^ | 0.02 | 8141241 | 266.2±28 | 234.9±28.7 | -31,3 | 9x10^-4^ | 3x10^-3^ |
| *SPATA18* | 4 | cg03103192 | TSS1500;N Shore | 60 ± 3 | 66 ± 4 | 6 | 3x10^-6^ | 0.01 | 8095021 | 123.4±25.5 | 113.1±27.4 | -10,3 | 3x10^-3^ | 0.01 |
| *STAT5A* | 17 | cg05117208 | TSS1500;N Shore | 50 ± 5 | 56 ± 4 | 6 | 2x10^-5^ | 0.01 | 8007212 | 491.3±68.9 | 432.6±60.2 | -58,7 | 2x10^-6^ | 4x10^-5^ |
| *SUPT16H* | 14 | cg12774429 | 3'UTR;TSS1500;S Shore | 74 ± 6 | 79 ± 3 | 5 | 1x10^-4^ | 0.02 | 7977674 | 837.5±79.1 | 745.8±99.1 | -91,7 | 6x10^-5^ | 4x10^-4^ |
| *SVIL* | 10 | cg17428896 | 5'UTR | 63 ± 4 | 69 ± 5 | 6 | 6x10^-5^ | 0.01 | 7932796 | 364.7±75.2 | 322.6±61.5 | -42,1 | 9x10^-3^ | 0.02 |
| *SYNJ2* | 6 | cg09667606 | Body;CpG Island | 67 ± 6 | 74 ± 5 | 7 | 2x10^-4^ | 0.02 | 8123006 | 74.2±13.1 | 68.2±10.7 | -6,0 | 3x10^-2^ | 0.04 |
| *SYNJ2* | 6 | cg11723077 | Body;S Shore | 58 ± 5 | 64 ± 4 | 6 | 1x10^-5^ | 0.01 | 8123006 | 74.2±13.1 | 68.2±10.7 | -6,0 | 3x10^-2^ | 0.04 |
| *SYTL3* | 6 | cg10296205 | 5'UTR | 43 ± 6 | 49 ± 4 | 6 | 6x10^-4^ | 0.03 | 8123080 | 95.1±16.8 | 83.3±15.4 | -11,8 | 3x10^-3^ | 0.01 |
| *TAF4* | 20 | cg03057951 | Body;CpG Island | 67 ± 6 | 72 ± 5 | 5 | 2x10^-3^ | 0.05 | 8067361 | 172.6±16.1 | 159.6±12.7 | -13,0 | 1x10^-3^ | 3x10^-3^ |
| *TEAD1* | 11 | cg23352146 | 5'UTR | 44 ± 5 | 50 ± 5 | 6 | 3x10^-6^ | 0.01 | 7938544 | 527.1±70.9 | 472.9±69.5 | -54,2 | 1x10^-3^ | 4x10^-3^ |
| *TFDP1* | 13 | cg02101742 | Body;CpG Island | 78 ± 6 | 85 ± 5 | 7 | 1x10^-3^ | 0.04 | 7970317 | 469.6±45.5 | 414.7±50.4 | -54,9 | 4x10^-5^ | 3x10^-4^ |
| *TIMP2* | 17 | cg12125614 | Body | 65 ± 6 | 70 ± 5 | 5 | 2x10^-4^ | 0.02 | 8018966 | 1741.3±318 | 1467.8±259.8 | -273,5 | 1x10^-4^ | 6x10^-4^ |
| *TIMP2* | 17 | cg12125614 | Body | 65 ± 6 | 70 ± 5 | 5 | 2x10^-4^ | 0.02 | 8018972 | 139.1±25.8 | 109.9±17.4 | -29,2 | 1x10^-6^ | 3x10^-5^ |
| *TNFSF13B* | 13 | cg01462856 | Body | 44 ± 6 | 49 ± 5 | 5 | 5x10^-5^ | 0.01 | 7969986 | 72.2±10.9 | 59.5±10.2 | -12,7 | 5x10^-6^ | 7x10^-5^ |
| *TNXB* | 6 | cg02270895 | Body | 77 ± 6 | 83 ± 3 | 6 | 2x10^-4^ | 0.02 | 8178712 | 501±188.8 | 395.2±111.4 | -105,8 | 4x10^-3^ | 0.01 |
| *TNXB* | 6 | cg02270895 | Body | 77 ± 6 | 83 ± 3 | 6 | 2x10^-4^ | 0.02 | 8125234 | 325.5±105.6 | 273.4±66.6 | -52,0 | 2x10^-2^ | 0.03 |
| *TRIM26* | 6 | cg08850243 | 5'UTR | 55 ± 5 | 60 ± 4 | 5 | 7x10^-5^ | 0.01 | 8124726 | 156.3±13.1 | 144.5±13.4 | -11,9 | 4x10^-4^ | 2x10^-3^ |
| *TRIM26* | 6 | cg08850243 | 5'UTR | 55 ± 5 | 60 ± 4 | 5 | 7x10^-5^ | 0.01 | 8179638 | 158.5±14 | 143.1±15.4 | -15,4 | 5x10^-5^ | 4x10^-4^ |
| *TRIM26* | 6 | cg15474894 | Body | 69 ± 6 | 75 ± 4 | 6 | 7x10^-4^ | 0.03 | 8124726 | 156.3±13.1 | 144.5±13.4 | -11,9 | 4x10^-4^ | 2x10^-3^ |
| *TRIM26* | 6 | cg15474894 | Body | 69 ± 6 | 75 ± 4 | 6 | 7x10^-4^ | 0.03 | 8179638 | 158.5±14 | 143.1±15.4 | -15,4 | 5x10^-5^ | 4x10^-4^ |
| *UBR2* | 6 | cg20646500 | Body;S Shelf | 43 ± 6 | 50 ± 6 | 7 | 3x10^-5^ | 0.01 | 8119529 | 727.2±71.9 | 676.7±74.1 | -50,5 | 1x10^-2^ | 0.02 |
| *WDR59* | 16 | cg00519320 | TSS1500;S Shore | 54 ± 6 | 60 ± 6 | 6 | 1x10^-3^ | 0.04 | 8002802 | 191.8±13.7 | 180.1±17.4 | -11,8 | 3x10^-3^ | 0.01 |
| *WIPF1* | 2 | cg15058645 | 5'UTR | 45 ± 5 | 50 ± 5 | 5 | 2x10^-4^ | 0.02 | 8056860 | 436.2±49.9 | 369.6±47.9 | -66,6 | 2x10^-7^ | 9x10^-6^ |
| *VPS52* | 6 | cg02024219 | Body | 64 ± 5 | 69 ± 6 | 5 | 3x10^-4^ | 0.02 | 8125649 | 394.9±27.3 | 377.9±30.3 | -17,0 | 1x10^-2^ | 0.02 |
| *VPS52* | 6 | cg02024219 | Body | 64 ± 5 | 69 ± 6 | 5 | 3x10^-4^ | 0.02 | 8180123 | 409±29.4 | 393.3±31 | -15,7 | 2x10^-2^ | 0.04 |
| *ZFHX3* | 16 | cg03878654 | 5'UTR;N Shore | 57 ± 7 | 66 ± 7 | 9 | 2x10^-4^ | 0.02 | 8002692 | 238±35.5 | 197.6±26.2 | -40,3 | 8x10^-7^ | 2x10^-5^ |
| *ZFHX3* | 16 | cg05516390 | 5'UTR;N Shelf | 42 ± 4 | 50 ± 4 | 8 | 1x10^-6^ | 4x10^-3^ | 8002692 | 238±35.5 | 197.6±26.2 | -40,3 | 8x10^-7^ | 2x10^-5^ |
| *ZMYM3* | X | cg19688321 | Body;TSS1500;S Shore | 56 ± 4 | 62 ± 4 | 6 | 3x10^-4^ | 0.02 | 8173457 | 128.1±15.3 | 112.6±14.7 | -15,5 | 8x10^-4^ | 3x10^-3^ |
| *ZNF385A* | 12 | cg07000334 | TSS1500;S Shore | 32 ± 5 | 38 ± 5 | 6 | 1x10^-5^ | 0.01 | 7963774 | 228.9±43.5 | 202.9±34.7 | -26,0 | 4x10^-3^ | 0.01 |
| *ZNF385A* | 12 | cg11856711 | TSS1500;S Shore | 21 ± 4 | 26 ± 4 | 5 | 2x10^-4^ | 0.02 | 7963774 | 228.9±43.5 | 202.9±34.7 | -26,0 | 4x10^-3^ | 0.01 |
| *ZNF385A* | 12 | cg17655624 | TSS1500;S Shore | 22 ± 6 | 28 ± 6 | 6 | 3x10^-4^ | 0.02 | 7963774 | 228.9±43.5 | 202.9±34.7 | -26,0 | 4x10^-3^ | 0.01 |
| *ZNF394* | 7 | cg25173392 | Body;N Shelf | 63 ± 5 | 69 ± 4 | 6 | 2x10^-4^ | 0.02 | 8141305 | 130.4±14.2 | 121.9±12.6 | -8,4 | 4x10^-2^ | 0.05 |

DNA methylation results are based on a paired non-parametric test whereas mRNA expression results are based on a paired t-test, and two-tailed *p*-values. Data are presented as mean ± SD.
