# Supplementary material for: A Six Months Exercise Intervention Influences the Genome-wide DNA Methylation Pattern in Human Adipose Tissue
Source: PLoS Genet. 2013 Jun 27;9(6):e1003572. doi: 10.1371/journal.pgen.1003572 (PMC3694844; doi:10.1371/journal.pgen.1003572)
Supplement: Table S4 — Assay design for technical validation of DNA methylation data using PyroSequencing. (DOC) [file pgen.1003572.s006.doc]

**Table S4.** Assay design for technical validation of DNA methylation data using PyroSequencing.

| **Infinium HumanMethylation450 BeadChip data** | | | **PyroSequencing validation assays (Qiagen)** | | | |
| --- | --- | --- | --- | --- | --- | --- |
| **Illumina probe ID** | **Position** | **Gene** | **Forward PCR primer** | **Reverse PCR primer** | **Sequencing primer** | **Pre-designed assays** |
| cg22077197 | chr2: 240143833 | *HDAC4* | 5'-GTATTTGGAGGGAGT AAGATTATTTG-3' | *bio*5'-ACCAAATTTTTATT TTCAACTCTTACTCAA-3' | 5'-GGGAGTAAGATT ATTTGTGT-3' (F) |  |
| cg10094994 | chr2: 240162323 | *HDAC4* | *bio*5'-AAAATAATGAAGA TGGGAAGTAAAGTA-3' | 5'-CCCTCTCAATATACA TCTAATCTACAA-3' | 5'-ATCTAATCTACA ACCCCAA-3' (R) |  |
| cg23880533 | chr12: 124896079 | *NCOR2* |  |  |  | Hs_cg23880533_01_PMC |
| cg21923525 | chr18: 9474143 | *RALBP1* |  |  |  | Hs_cg21923525_01_PMC |

*bio*, biotinylated primer; (F), forward strand; (R), reverse strand
