# Supplementary material for: A Six Months Exercise Intervention Influences the Genome-wide DNA Methylation Pattern in Human Adipose Tissue
Source: PLoS Genet. 2013 Jun 27;9(6):e1003572. doi: 10.1371/journal.pgen.1003572 (PMC3694844; doi:10.1371/journal.pgen.1003572)
Supplement: Table S5 — siRNA assays. (DOC) [file pgen.1003572.s007.doc]

**Table S5.** siRNA assays.

| **siRNA assays** | **Sense strand** | **Anti sense strand** |
| --- | --- | --- |
| Hdac4 MSS209678 | GAGCAGCAGAGGAUCCACCAGUUAA | UUAACUGGUGGAUCCUCUGCUGCUC |
| Hdac4 MSS209679 | CACCGGAACCUGAACCACUGCAUUU | AAAUGCAGUGGGUUCAGGUUCCGGUG |
| Hdac4 MSS209680 | GCAACGUCAGCACUGAGAAUGGCAU | AUGCCAUUCUCAGUGCUGACGUUGC |
| Ncor2 MSS209219 | GACCGAGGAAGAGCCUGAAGACAAA | UUUGUCUUCAGGCUCUUCCUCGGUC |
| Ncor2 MSS209220 | CGAGACAAGUCCAUCCUCACGUCUA | UAGACGUGAGGAUGGACUUGUCUCG |
| Ncor2 MSS209221 | GGAGCAGGCCUUAUGACCUGUAGAA | UUCUACAGGUCAUAAGGCCUGCUCC |
